# The Efficacy of Traditional Chinese Exercises in Patients With Chronic Heart Failure: An Umbrella Review and Meta-Analysis

**DOI:** 10.31083/RCM46055

**Published:** 2026-03-20

**Authors:** Xiaoyu Zhao, Rongjun Zou, Haoran Miao, Xing Chang, Kroekkiat Chinda, Fang Chen, Miao Zhang, Jin Zhuo, Xuejing Sun, Yijun Chen, Chao Li, Qingyong He, Cheng Luo, Timothy Kwok, Dachun Xu, Yiqian Zhang, Hao Zhou, Xiaoping Fan, Sang-Bing Ong

**Affiliations:** ^1^Department of Medicine and Therapeutics, Faculty of Medicine, Chinese University of Hong Kong (CUHK), Hong Kong, China; ^2^Kunming Institute of Zoology-The Chinese University of Hong Kong (KIZ-CUHK) Joint Laboratory of Bioresources and Molecular Research of Common Diseases, Hong Kong, China; ^3^Department of Cardiovascular Surgery, Guangdong Provincial Hospital of Chinese Medicine, The Second Affiliated Hospital of Guangzhou University of Chinese Medicine, The Second Clinical College of Guangzhou University of Chinese Medicine, 510120 Guangzhou, Guangdong, China; ^4^Department of Thoracic Cardiovascular Surgery, The Eighth Affiliated Hospital of Sun Yat-sen University, 518033 Shenzhen, Guangdong, China; ^5^Department of Cardiology, Guang’anmen Hospital of Chinese Academy of Traditional Chinese Medicine, 100053 Beijing, China; ^6^Department of Physiology, Faculty of Medical Science, Naresuan University, 65000, Phitsanulok, Thailand; ^7^College of Pharmacy, Guangzhou University of Chinese Medicine, 510405 Guangzhou, Guangdong, China; ^8^School of Pharmaceutical Sciences, Guangzhou University of Chinese Medicine, 510006 Guangzhou, Guangdong, China; ^9^College of Traditional Chinese Medicine, Shandong University of Traditional Chinese Medicine, 250355 Jinan, Shandong, China; ^10^Department of Cardiovascular Surgery, The First Affiliated Hospital of Guangxi Medical University, 530021 Nanning, Guangxi, China; ^11^Department of Cardiology, Shanghai Tenth People’s Hospital, Tongji University School of Medicine, 200072 Shanghai, China; ^12^Senior Department of Cardiology, Chinese People’s Liberation Army General Hospital, Medical School of Chinese People’s Liberation Army, 100853 Beijing, China; ^13^Hong Kong Hub of Paediatric Excellence (HK HOPE), Hong Kong Children’s Hospital (HKCH), Kowloon Bay, Hong Kong, China

**Keywords:** heart failure, exercise therapy, cardiac rehabilitation, Tai Chi, Qigong, traditional chinese medicine, meta-analysis as topic

## Abstract

**Background:**

Chronic heart failure (CHF) is a common clinical syndrome characterized by reduced exercise capacity, diminished quality of life (QoL), and unfavorable cardiovascular outcomes. Conventional cardiac rehabilitation often requires moderate-to-high-intensity exercise, which may be tolerated poorly by many CHF patients. Low-intensity mind–body interventions, such as traditional Chinese exercises (TCEs), are potentially more suitable; however, the evidence from existing studies is fragmented and sometimes inconsistent. Thus, this study aimed to conduct an umbrella review of systematic reviews (SRs) and meta-analyses (MAs) to evaluate the effectiveness of TCEs in improving exercise capacity, QoL, and cardiovascular function in patients with CHF.

**Methods:**

An umbrella review of SRs/MAs was conducted by searching English and Chinese databases without language limits and focusing on randomized controlled trials (RCTs) that assessed the additional benefit of TCEs in individuals with CHF. Methodological quality was appraised using the A Measurement Tool to Assess Systematic Reviews 2 (AMSTAR 2) checklist and the Risk of Bias in Systematic Reviews (ROBIS) instrument. The Grading of Recommendations Assessment, Development, and Evaluation (GRADE) system was utilized to quantify the certainty of evidence. Individual trial data were retrieved, and re-meta-analyses were performed using standard statistical procedures, with publication bias assessed via Egger's test.

**Results:**

A total of 15 SRs/MAs were included, encompassing 65 original trials. Our re-meta-analysis indicated that TCEs were associated with substantially longer 6-minute walk test (6-MWT) values, improved QoL measured by the Minnesota Living with Heart Failure Questionnaire (MLHFQ), higher left ventricular ejection fraction (LVEF), reduced B-type natriuretic peptide (BNP) levels, and enhanced maximal oxygen consumption (VO_2_max). Baduanjin exhibited a particularly robust effect on lowering N-terminal pro-B-type natriuretic peptide (NT-proBNP) concentrations, while Yijinjing yielded comparatively greater improvements in VO_2_max. Nonetheless, limitations such as suboptimal methodological quality and overlapping study samples require cautious interpretation.

**Conclusions:**

TCEs may serve as a beneficial adjunct to standard care for CHF, improving exercise capacity, QoL, and key cardiac markers. Large, rigorous RCTs with extended follow-up are needed to confirm the durability of TCEs and further define the role of these exercises in comprehensive CHF rehabilitation.

**The PROSPERO Registration:**

CRD420251003129 (https://www.crd.york.ac.uk/PROSPERO/view/CRD420251003129).

## 1. Introduction

Chronic heart failure (CHF) is a complex, progressive, and refractory clinical syndrome that typically results from structural or functional cardiac abnormalities of the heart, manifesting in the form of impaired ventricular ejection and/or filling and represents the terminal stage of many cardiovascular diseases [1, 2]. More than 64 million people worldwide are affected by CHF, which results in dyspnea, reduced exercise tolerance, diminished quality of life (QoL), and even secondary organ dysfunction, such as renal impairment. According to recent data [3, 4], the prevalence of CHF among adults is about 1% to 2%, but this can rise to nearly 10% in those aged over 70 years. Meanwhile, owing to an aging population and improved diagnostic accuracy, the prevalence of CHF is expected to continue increasing [5, 6, 7].

Conventional treatments, such as pharmacotherapy and lifestyle changes, aim to alleviate symptoms, improve function, and reduce hospitalizations [2, 8, 9]. However, the multifaceted nature of CHF necessitates adjunctive approaches to enhance patient outcomes [10]. Traditional exercise-based rehabilitation programs usually emphasize high-intensity physical activity [11, 12], which may not be suitable for all patients. Individuals with CHF frequently have limited physical capacity, making such demanding programmes challenging to perform. Consequently, there is growing interest in alternative, low-impact exercise modalities that can be safely and effectively integrated into the routine care of CHF patients [13, 14].

Therefore, traditional Chinese exercises (TCEs), which include Tai Chi, Baduanjin, Yijinjing, and Liuzijue, have attracted increasing attention as potential complementary therapies for CHF. Rooted in ancient Chinese philosophy and medicine, TCEs blend gentle movements, breath regulation, and mindful focus, which are practised consistently to foster holistic well-being. Additionally, the low-intensity and rhythmic nature of these exercises makes TCEs particularly suitable for individuals with reduced physical capabilities. Each form offers distinct advantages: Tai Chi, originating from martial arts, focuses on balance, coordination, and fluid transitions—often referred to as “moving meditation”; Baduanjin provides simple, accessible stretching movements aimed at stimulating organ meridians; Yijinjing combines dynamic motions with stretching to enhance strength, flexibility, and resilience; Liuzijue employs specific vocalized sounds and breathwork to harmonize organ function. While Tai Chi is more structured, with choreographed sequences, Qigong (including Baduanjin, Yijinjing, and Liuzijue) is generally more flexible and energy-focused. Nonetheless, both are effective for CHF patients and can be tailored to individual needs, making these exercises valuable options for enhancing physical and mental health in this population.

Emerging evidence indicates that TCEs may also confer cardiovascular benefits, including improved exercise capacity, enhanced QoL, and reduced psychological distress [15, 16, 17, 18]. However, the consistency of these benefits across trials remains unclear due to substantial variation in study design, intervention protocols, and outcome measures. Notably, no current study has provided a consolidated, head-to-head appraisal of this fragmented evidence while simultaneously re-analysing the underlying randomized controlled trials (RCTs).

Therefore, the present work provides several key innovations. First, to our knowledge, this study provides the first umbrella review to compile all available systematic reviews (SRs)/meta-analyses (MAs) on TCEs for CHF and quantify review overlap using the Graphical Representation Of Overlap for OVErviews (GROOVE) tool. Second, this study applies a comprehensive quality-assessment framework to judge both review rigour and evidence certainty: Methodological quality was appraised with A Measurement Tool to Assess Systematic Reviews 2 (AMSTAR 2), risk of bias was evaluated with the Risk of Bias in Systematic Reviews (ROBIS) tool, reporting quality was measured against the Preferred Reporting Items for Systematic Reviews and Meta-Analyses (PRISMA) 2020 checklist and certainty of evidence for every pooled outcome was determined with the Grading of Recommendations Assessment, Development and Evaluation (GRADE) approach. Third, we conducted a de-duplicated re-meta-analysis of data from individual RCTs, thereby producing the most up-to-date and least biased pooled estimates. Crucially, by directly re-analysing individual RCT data, we can minimise the impact of overlapping reviews, obtain more reliable effect estimates, highlight research gaps, and provide clearer guidance on the role of TCEs in CHF rehabilitation.

## 2. Materials and Methods

This umbrella review was conducted in accordance with PRISMA guidelines. This study is registered in the International Prospective Register of Systematic Reviews.

### 2.1 Eligibility Criteria 

The eligibility criteria were developed according to the population, intervention, comparator, outcomes, and study design (PICOS) framework. We considered SRs/MAs that synthesised evidence exclusively from RCTs investigating TCEs for the management of CHF. Adult patients (≥18 years) with any etiology or New York Heart Association (NYHA) classification of heart failure were eligible, including the full spectrum of phenotypes associated with left ventricular ejection fraction (LVEF): heart failure with reduced (HFrEF, LVEF <40%), mildly reduced (HFmrEF, LVEF 40–49%), and preserved ejection fraction (HFpEF, LVEF ≥50%). Reviews reporting patients treated either during hospitalisation or after discharge were admissible. Acceptable interventions comprised Tai Chi, Baduanjin, Qigong, Liuzijue, Yijinjing, or other clearly defined TCEs administered alone or as an adjunct to conventional care; eligible comparators included usual care, aerobic or resistance exercise, pharmacotherapy, or no-exercise control. Reviews had to provide quantitative results for at least one pre-specified endpoint, exercise capacity (*e*.*g*., 6-minute walk test (6-MWT) (m) or VO_2_max (mL⋅kg^-1^⋅min^-1^)), cardiac function parameters (*e*.*g*., LVEF (%), plasma B-type natriuretic peptide (BNP) (pg/mL) or N-terminal pro-B-type natriuretic peptide (NT-proBNP) (pg/mL)), health-related QoL, or clinical outcomes such as hospitalisation or mortality. We excluded reviews whose primary studies were not RCTs, those in which CHF-specific outcomes were absent, protocol-only publications, and reviews that failed to report extractable data (number of participants, effect size, and 95% confidence interval (CI)).

### 2.2 Search Strategy

A comprehensive literature search was conducted in the Cochrane Library, PubMed (MEDLINE), Embase, Web of Science Core Collection, and four major Chinese databases (China National Knowledge Infrastructure, SinoMed, Wanfang Data, and China Science and Technology Journal Database) from inception to 31 March 2025. The strategy combined controlled vocabulary (MeSH/Emtree terms such as “heart failure”, “Tai Chi”) with free-text keywords including “chronic heart failure,” “Tai Chi,” “Baduanjin,” “Liuzijue,” “Yijinjing,” “meta-analysis,” and “systematic review.” Search algorithms were adapted to the syntax of each platform, and the complete strategies are reproduced in **Supplementary Table 1**. In addition, the reference lists of all eligible reviews and relevant clinical trial or systematic review registries (ClinicalTrials.gov, PROSPERO, INPLASY) were manually screened to identify additional citations. No restrictions were placed on language, publication date, or geographical origin; potentially relevant grey literature (theses, conference proceedings, preprints) was also considered. Two reviewers conducted the searches independently, and a third reviewer reconciled any discrepancies in retrieval or database-specific adaptations.

### 2.3 Data Extraction and Analysis 

All records retrieved from the eight prespecified databases were exported to EndNote software (Version X9; Clarivate Analytics, Philadelphia, PA, USA) and de-duplicated both automatically and manually. The full search strategies for each database are available in **Supplementary Table 1**. Two trained reviewers independently screened titles and abstracts, then assessed the full texts against the eligibility criteria; any disagreements were resolved by consensus, and when necessary, by a third senior reviewer.

Before formal extraction, a pilot-tested, standardised form was developed. Two reviewers independently extracted the following information: (1) general study characteristics (first author, year, country); (2) number of included RCTs and total participants; (3) population details and intervention/control features; (4) risk-of-bias tool applied; (5) primary outcomes and provided conclusions; (6) numerical results of each original RCT. A second pair of reviewers cross-checked all extracted data, and remaining discrepancies were settled in consultation with an experienced methodological adjudicator.

Given the inconsistent effect values across MAs/SRs, we also extracted RCT data from each article, pooled the outcome metrics, and re-estimated the meta-analysis. Heterogeneity between studies was assessed and quantified using the Q and I^2^ statistics, with a fixed-effects model if no heterogeneity was present and a random-effects model if heterogeneity was present. Subgroup analyses were performed to explore sources of heterogeneity. We also used Eggers’s and funnel plot tests to detect publication bias and conducted sensitivity analyses to assess the robustness of the results. Statistical analyses were performed using R software (Version 4.1.1; R Foundation for Statistical Computing, Vienna, Austria), with *p* < 0.05 considered statistically significant.

### 2.4 Quality Assessment

For methodological appraisal, two reviewers independently applied four validated instruments (AMSTAR 2, ROBIS, PRISMA 2020, and GRADE). A senior reviewer adjudicated disagreements, and an independent methodological consultant confirmed the final ratings.

AMSTAR 2 [19] assesses sixteen items, of which items 2, 4, 7, 9, 11, 13, and 15 are critical domains. Individual items were categorised as “yes,” “partial yes,” or “no,” and the established decision algorithm generated an overall confidence judgement of high, moderate, low, or critically low.

ROBIS [20] involves three sequential phases. After confirming the match between the review question and our own, four domains, study eligibility criteria, identification and selection of studies, data collection and appraisal, and synthesis and findings, were examined by responding to signalling questions. Each domain and the overall review were rated as low, high, or unclear risk.

PRISMA 2020 [21] compliance was evaluated across 27 items, with a score of 1 for fully reported items and 0 for absent or insufficient reporting, yielding a total score of 0 to 27; higher scores indicate more complete reporting.

GRADE [22, 23] rates the certainty of evidence for each pooled outcome. As all pooled estimates originated from RCTs, evidence was categorized as high and then downgraded one level for each serious concern identified in the domains of risk of bias, inconsistency, indirectness, imprecision, or publication bias. The resulting certainty categories were then classified as high, moderate, low, or very low.

### 2.5 Evaluating Literature Overlap

The corrected covered area (CCA) quantifies the degree of overlap in the included literature [24]. An excessive CCA can occur when different SRs/MAs include many of the same RCTs, owing to similar search strategies, a concentrated evidence base, and the limited number of available trials. Such redundancy may bias results and diminish the reliability of conclusions [25, 26, 27].

We employed the GROOVE tool (v1.0; developed by Javier Bracchiglione and colleagues at the Interdisciplinary Centre for Health Studies (CIESAL), Universidad de Valparaíso, Chile, and Iberoamerican Cochrane Centre, Spain; freely available at http://doi.org/10.17605/OSF.IO/U2MS4 and https://es.cochrane.org/es/groovetool) [28] to graph the degree of overlap and calculate CCA; the CCA was calculated using the formula as CCA = (N – r)/[(r × c) – r]. The subsequent results were classified into low overlap (0–5%), moderate overlap (6–10%), high overlap (11–15%), and very high overlap (>15%) [29].

### 2.6 Equity, Diversity, and Inclusion Statement

Our team comprises male and female researchers across multiple disciplines, including Western medicine practitioners and traditional Chinese medicine (TCM) physicians. Since most of the included studies were conducted in China, geographical diversity is limited; this limitation is acknowledged in the Discussion. This review did not analyse the influence of socioeconomic status or race/ethnicity on outcomes. We also discuss how cultural context may affect the application of TCEs in the Discussion.

## 3. Results

### 3.1 Search Results

**Fig. 1.**
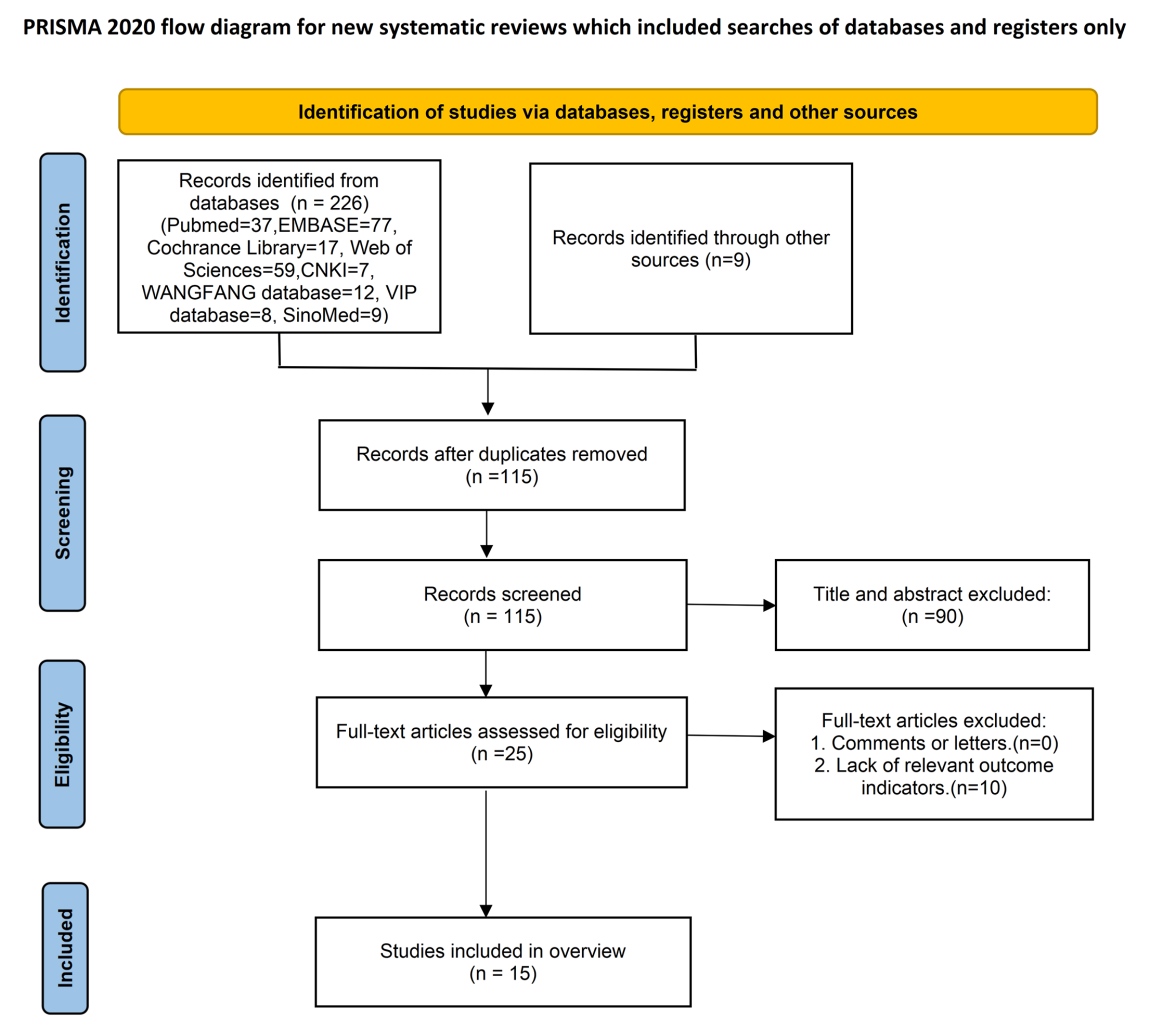
**Flow diagram of the literature selection process**.

The search retrieved 235 records in total, 226 from electronic databases and 9 from supplementary sources. After de-duplication, 115 citations remained. Title-and-abstract screening eliminated 90 records, leaving 25 articles for full-text assessment. 10 of these were excluded because the articles lacked the relevant outcome indicators; 15 studies [30, 31, 32, 33, 34, 35, 36, 37, 38, 39, 40, 41, 42, 43, 44] were ultimately included in the overview. The screening workflow is shown in Fig. 1, and details of the excluded studies appear in **Supplementary Table 2**.

### 3.2 Characteristics of Included SRs/MAs

The 15 included SRs/MAs were published between 2013 and 2023; 14 originated in China, while one was from the United States. Three papers were written in Chinese, whereas 12 were in English. The number of RCTs per review ranged from 4 to 41, and the sample size ranged from 229 to 3209 participants. In the controlled comparisons, the experimental group received conventional treatment augmented with TCEs (Tai Chi or Qigong variants such as Baduanjin, Liuzijue, and Yijinjing). In contrast, the control group received traditional care alone (medication, health education, routine nursing), with or without additional strength training. Quality assessment methods included Cochrane criteria (13 studies), Downs and Black index (one study), and the modified Jadad scale (one study), reflecting methodological diversity. Table 1 (Ref. [30, 31, 32, 33, 34, 35, 36, 37, 38, 39, 40, 41, 42, 43, 44]) provides a detailed overview of the study characteristics.

**Table 1.** **Characteristics of the included SRs/MAs**.

Author, year (Country)	Trials (participants)	Intervention group	Control group	Quality assessment	Main results
Yang *et al*. [30], 2023 (CHN)	8 (468)	Baduanjin + conventional treatment	Conventional treatment	Cochrane	Baduanjin is a safe, feasible, and acceptable intervention, improving QoL and exercise capacity in HF patients. More rigorous RCTs are needed for rehabilitation.
Mei e*t al*. [31], 2023 (CHN)	15 (1180)	Baduanjin + conventional treatment	Conventional treatment	Physiotherapy evidence database	Meta-analysis supports the benefits of Baduanjin on QoL, cardiac function, and VO_2_max in Chinese CHF patients. Widespread recommendations require further rigorous, larger studies.
Dai *et al*. [32], 2023 (CHN)	21 (1665)	TCEs + conventional treatment	Conventional treatment	Cochrane	TCEs improved various recovery, clinical, QoL, and physiological indicators in CHF. While expanding participant application is valuable, existing evidence is insufficient and highly heterogeneous, necessitating more high-quality trials.
Bao *et al*. [33], 2023 (CHN)	41 (3209)	TCEs + conventional treatment	Conventional treatment	Cochrane	TCEs enhance exercise capacity, cardiac function, and QoL in CHF, potentially serving as an optimal, accessible exercise-based cardiac rehabilitation approach.
Hui *et al*. [34], 2022 (CHN)	15 (1236)	Tai Chi + conventional treatment	Conventional treatment	Cochrane	Tai Chi appears safe and beneficial for the health status of patients with CHF. However, further high-quality, long-term studies are needed for a comprehensive evaluation.
Yao *et al*. [35], 2021 (CHN)	9 (721)	TCEs + conventional treatment	Conventional treatment	Cochrane	TCEs show potential in improving cardiac function, motor function, and QoL. TCEs may be an effective adjuvant therapy for HF, but further rigorous studies are urgently needed to confirm these results, given the inclusion of low-quality evidence.
Taylor-Piliae and Finley [36], 2020 (USA)	6 (229)	Tai Chi + conventional treatment	Conventional treatment	The modified Downs and Black Quality Index checklist	Tai Chi can be easily integrated into existing cardiac rehabilitation programs. Broader recommendations depend on additional rigorous studies with larger samples.
Liao *et al*. [38], 2020 (CHN)	22 (1646)	TCEs + conventional treatment	Conventional treatment	Cochrane	TCEs may benefit the prognosis of CHF patients and appear relatively safe. Intervention intensity and follow-up time seem to influence effects. However, further rigorous studies are urgently needed due to low-quality evidence.
Chen *et al*. [37], 2020 (CHN)	33 (2465)	Tai Chi and Qigong + conventional treatment	Conventional treatment	Cochrane	Tai Chi and Qigong practices are promising, low-cost rehabilitation therapies with multiple physical benefits, suitable as an adjunct or alternative to conventional exercises, especially for home-based settings.
Wang and Ma [42], 2020 (CHN)	7 (543)	Baduanjin + conventional treatment	Conventional treatment	Cochrane	Baduanjin can improve cardiac function in HF patients and is utilized for stable rehabilitation treatment.
Li *et al*. [43], 2018 (CHN)	7 (446)	Tai Chi + conventional treatment	Conventional treatment	Cochrane	Tai Chi training significantly improves heart function and QoL for HF patients, making this exercise applicable to stable HF rehabilitation.
Wei *et al*. [44], 2017 (CHN)	10 (689)	Tai Chi + conventional treatment	Conventional treatment	Cochrane	Current evidence suggests Tai Chi is feasible for HF patients, showing positive effects on QoL and physiological functions. However, due to limited study quality and quantity, these conclusions require validation by more high-quality studies.
Ren *et al*. [39], 2017 (CHN)	11 (656)	Tai Chi + conventional treatment	Conventional treatment	Cochrane	Tai Chi could improve 6-MWT, QoL, and LVEF in HF patients, and may reduce BNP levels and heart rate. Nevertheless, evidence of its impact on other important long-term clinical outcomes is lacking, necessitating larger, more sustainable RCTs.
Gu *et al*. [40], 2017 (CHN)	13 (918)	Tai Chi + conventional treatment	Conventional treatment	Cochrane	Despite heterogeneity and risk of bias, this meta-analysis confirms Tai Chi as a potentially effective cardiac rehabilitation method for CHF. Nonetheless, larger, well-designed RCTs are needed to address bias.
Pan *et al*. [41], 2013 (CHN)	4 (242)	Tai Chi + conventional treatment	Conventional treatment	Jadad	Tai Chi may improve QoL in CHF patients and could be considered for cardiac rehabilitation. However, evidence for its effect on other important clinical outcomes is currently lacking, and further larger RCTs are urgently needed.

**Abbreviations:** 6-MWT, 6-minute walk test; BNP, B-type natriuretic peptide; CHF, chronic heart failure; CHN, China; conventional treatment, including health education, usual care, pharmacologic therapy, dietary, and endurance training; HF, heart failure; LVEF, left ventricular ejection fraction; QoL, quality of life; RCT, randomized controlled trial; SRs/MAs, systematic reviews and meta-analyses; TCEs, traditional Chinese exercises; USA, United States of America; VO_2_max, maximal oxygen consumption.

**Fig. 2.**
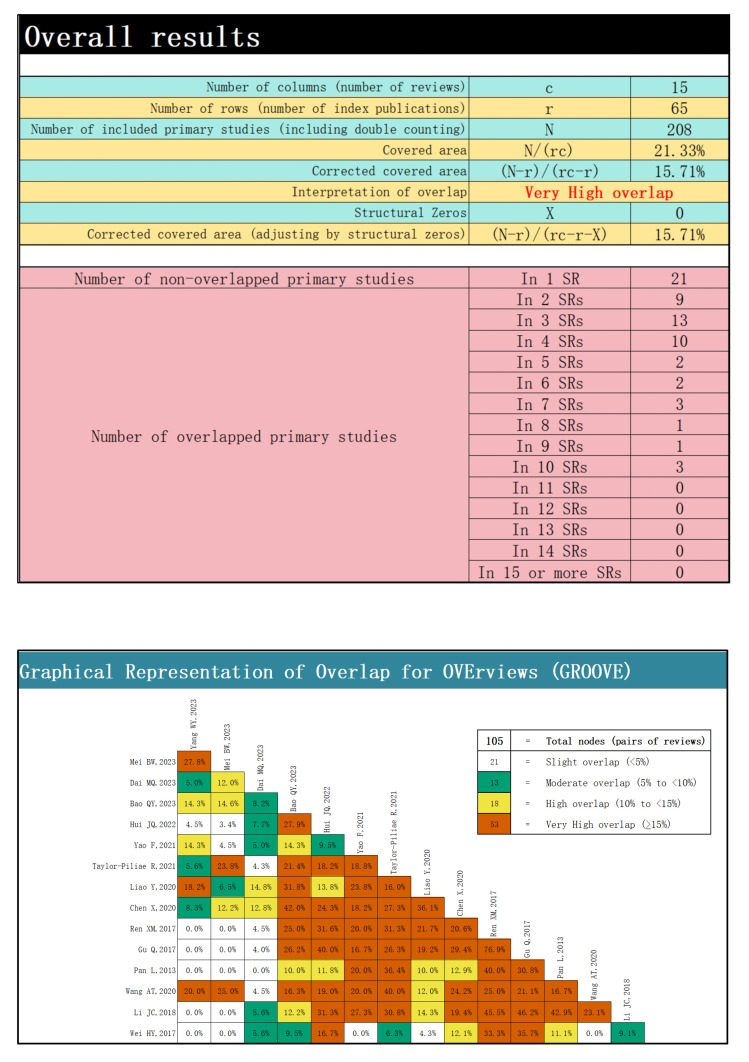
**Overlap analysis of the included reviews (GROOVE tool)**. GROOVE, Graphical Representation of Overlap for OVErviews.

From the 15 SRs/MAs, we extracted 65 unique RCTs; detailed cross-links appear in **Supplementary Table 3**. The GROOVE tool provided the evidence matrix, and the CCA was calculated to measure the degree of overlap. The results showed an adjusted CCA of 15.71%, indicating a very high degree of overlap between the reviews (Fig. 2). This redundancy suggests that many analyses relied on the same core trials or employed similar search strategies, potentially limiting analytical diversity and weakening the robustness of pooled conclusions.

### 3.3 Methodological Quality Assessment

The AMSTAR 2 appraisal revealed that 14 SRs/MAs were rated with a critically low quality, while one was rated low quality. Recurring problems included poorly described methods, absence of a registered protocol, and no prespecified plan for exploring heterogeneity. In addition, most reviews did not provide a list of excluded studies with justifications, and a few failed to declare the associated funding sources or conflicts of interest. Detailed AMSTAR 2 scores are provided in **Supplementary Table 4**.

### 3.4 ROBIS Assessment

The ROBIS evaluation indicated that all reviews were at low risk of bias for domain 1 (the relevance of the research question and the appropriateness of the eligibility criteria). By contrast, every review was judged to be at high risk in domain 2 because the search strategy was not comprehensive; most relied solely on database queries without additional methods or language restrictions. For domain 3, 14 reviews were at low risk; for domain 4, 11 were at low risk. In the overall judgement, 11 reviews were considered to have a low risk of bias. The full ROBIS ratings are presented in **Supplementary Table 5**.

### 3.5 Reporting Quality Assessment

Compliance with the PRISMA 2020 checklist is summarised in **Supplementary Table 6**. The title (item 1) and introduction (items 3–4) were fully reported (100%), and the abstract (item 2) was almost complete at 93.33%. Within the methods section, most items were adequately covered; however, the search strategy (item 7) and synthesis methods (item 13f) were covered at only 73.33% and 66.67%, respectively. Risk-of-bias assessment (item 11) and reporting bias assessment (item 14) achieved scores of 86.67% and 80.00%, respectively. In the results section, completeness was generally high, but study selection (item 16b, 0%) and certainty of evidence (item 22, 26.67%) were poorly addressed. The discussion was largely complete. In the information section, registration/protocol (item 24, 0–33.33%), support (item 25, 60.00%), competing interests (item 26, 53.33%), and data/code availability (item 27, 40.00%) showed lower reporting rates.

### 3.6 Evidence Quality Assessment

**Supplementary Table 7** presents the GRADE-based evaluation of 83 outcomes from 15 SRs/MAs. Among these, 46 outcomes were graded critically low quality, 28 low quality, and 9 moderate quality; none received a high rating. Since blinding was seldom feasible in the included RCTs, all outcomes were downgraded for study limitations. Substantial and unexplained heterogeneity affected 61 outcomes, resulting in a downgrading for inconsistency. Imprecision was identified in 51 outcomes due to small sample sizes and wide CIs. Publication bias was suspected in 28 outcomes, as suggested by asymmetric or absent funnel plots. Collectively, these issues markedly reduced the overall certainty of the evidence.

These methodological weaknesses and the marked overlap between reviews underscore the need for our re-meta-analysis to provide a clearer and more reliable evidence base.

### 3.7 Combined Results From Published Meta-Analyses and Re-Meta-Analyses

**Fig. 3.**
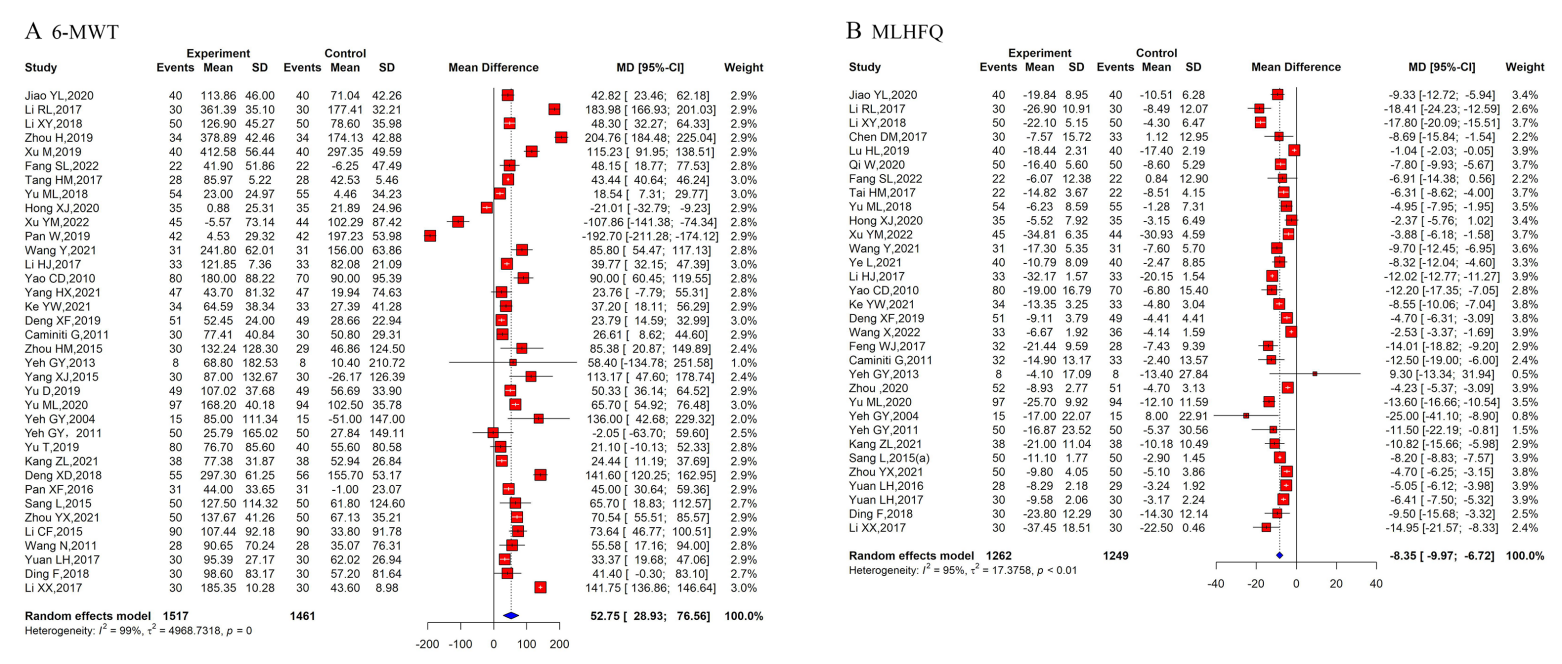
**Pooled effect sizes of the included outcomes**. (A) 6-MWT. (B) MLHFQ. 6-MWT, 6-minute walk test; MLHFQ, Minnesota Living with Heart Failure Questionnaire.

**Fig. 4.**
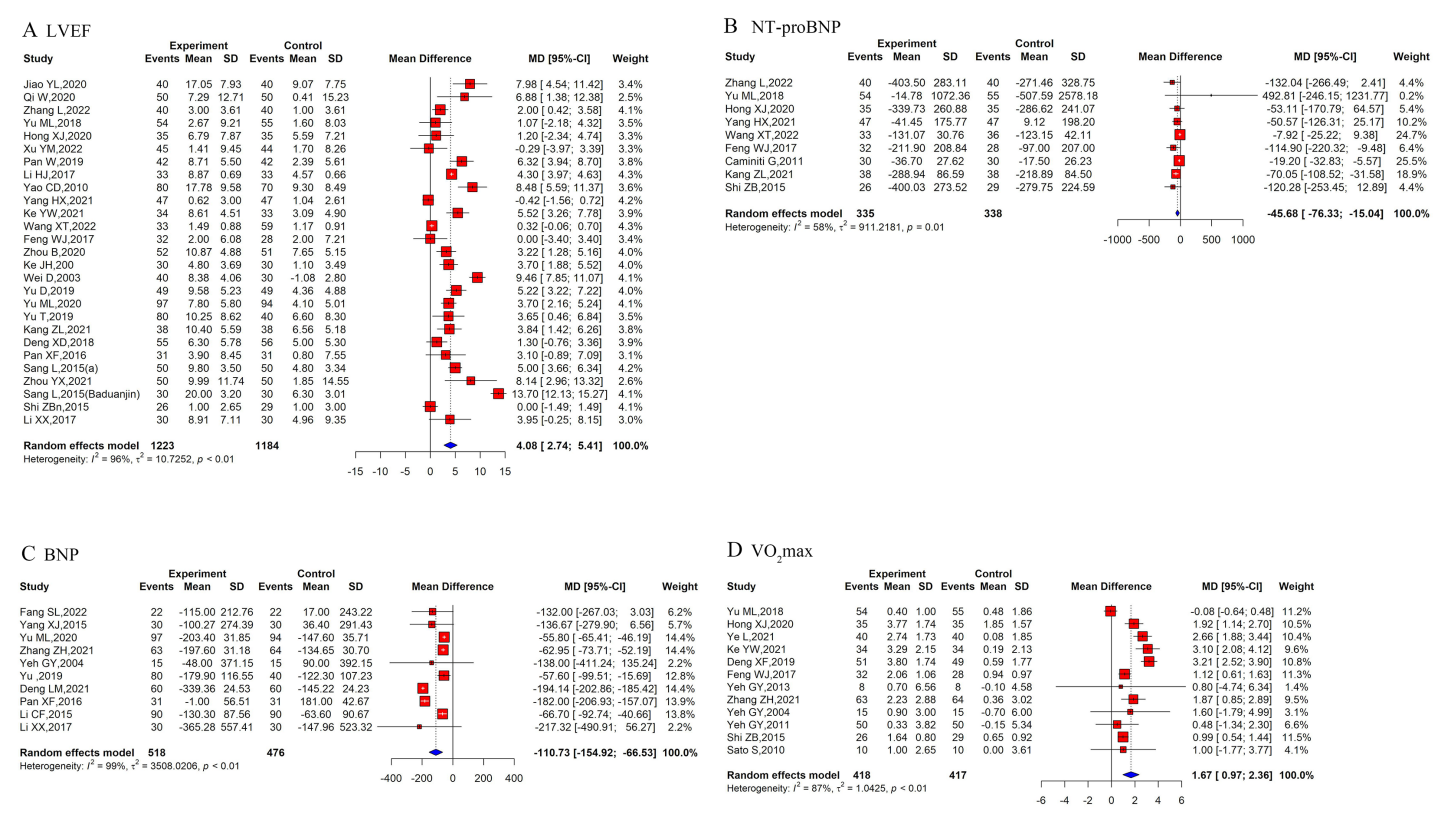
**Repooled effect sizes of cardiac function and exercise capacity outcomes**. (A) LVEF. (B) NT-proBNP. (C) BNP. (D) VO_2_max. LVEF, left ventricular ejection fraction; NT-proBNP, N-terminal pro-B-type natriuretic peptide; BNP, B-type natriuretic peptide; VO_2_max, maximal oxygen uptake.

This section presents the synthesised findings from both published SRs/MAs and our re-meta-analysis of individual RCTs. In this umbrella review, we reassessed the outcome indicators included in SRs/MAs, with detailed re-pooled effect sizes for each outcome (Figs. 3 and 4).

#### 3.7.1 6-MWT

Published systematic reviews and meta-analyses [30, 32, 33, 35, 38, 39, 40, 42, 43, 44] consistently indicate that TCEs significantly prolong 6-MWT distance compared to controls, suggesting enhanced exercise capacity. Three reviews specifically on Baduanjin reported highly significant improvements (all *p* < 0.001) [31, 33, 42]. While Dai *et al*. [32] found that performing TCEs for longer than three months yielded better effects, Hui *et al*. [34] noted no significant difference between 6-month and 12-month Tai Chi interventions. Taylor-Piliae and Finley [36] suggested a slight beneficial effect, although one small sample review (four RCTs) reported no significant improvement from Tai Chi [41].

Our re-meta-analysis, which pooled 36 original RCTs, confirmed these findings. The group that performed TCEs demonstrated a significantly greater 6-MWT distance (random-effects mean difference (MD) = 52.75 m, 95% CI: 28.96–76.56; Fig. 3A), despite high heterogeneity (*p* = 0; I^2^ = 99%). Subgroup analyses showed no significant differences among Baduanjin (MD = 50.99 m, 95% CI: 1.77–100.21), Tai Chi (MD = 51.33 m, 95% CI: 34.20–68.46), Yijinjing (MD = 37.20 m, 95% CI: 18.11–59.26), Liuzijue (MD = 85.38 m, 95% CI: 20.87–149.89), and mixed exercises (MD = 52.75 m, 95% CI: 28.93–76.56).

Both published meta-analyses and our re-meta-analysis consistently support that TCEs enhance functional exercise capacity, as measured by the 6-MWT.

#### 3.7.2 Minnesota Living With Heart Failure Questionnaire (MLHFQ) Scores

Systematic reviews and meta-analyses [30, 31, 32, 33, 34, 35, 37, 38, 39, 40, 41, 42, 43, 44] have consistently reported that performing TCEs results in significant improvements in MLHFQ scores. Dai *et al*. [32] indicated that longer TCE durations yielded better QoL outcomes, with subgroup analysis confirming greater improvements for interventions >3 months. Hui *et al*. [34] reported significant QoL improvements after 3 and 6 months of Tai Chi, with greater gains at 6 months. Taylor-Piliae and Finley [36] reported that Tai Chi significantly improved QoL in patients with heart failure, with effects comparable to those of other exercise interventions (Hedges g = 0.617, *p* = 0.000, I^2^ = 0%).

Our re-meta-analysis, based on 32 original RCTs, similarly found significant improvements. The TCEs group had significantly lower MLHFQ scores than controls (random-effects MD = –8.35, 95% CI: –9.97 to –6.72; Fig. 3B), suggesting a substantial QoL advantage. High heterogeneity was also observed (*p* < 0.01; I^2^ = 95%). The subgroup analyses revealed no significant differences among the Baduanjin (MD = –7.95, 95% CI: –10.32 to –5.60), Tai Chi (MD = –8.89, 95% CI: –11.34 to –6.43), and Yijinjing groups (MD = –8.35, 95% CI: –9.97 to –6.72) (*p* = 0.56).

Both sets of analyses consistently demonstrated that TCEs significantly improve the QoL for patients with heart failure.

#### 3.7.3 LVEF

Multiple systematic reviews and meta-analyses [31, 32, 33, 39, 40, 42, 43, 44] have demonstrated that TCEs significantly increase LVEF values. However, one study [32] reported no significant difference in outcomes between intervention durations of less than 3 months and those of more than 3 months (*p* = 0.14 and *p* = 0.16, respectively). Yao *et al*. [35] also showed no significant improvement in LVEF with TCEs compared to controls (MD = 1.38, 95% CI: [–3.08, 5.84]; *p* = 0.54). Liao *et al*. [38], despite overall significant results, highlighted non-overlapping CIs.

Our re-meta-analysis, which pooled 27 RCTs, similarly found significant improvements. TCEs significantly increased LVEF (random-effects MD = 4.08, 95% CI: 2.74–8.15; Fig. 4A), despite substantial heterogeneity (*p* < 0.01; I^2^ = 96%). The subgroup analyses revealed no significant differences among the Baduanjin (MD = 3.42, 95% CI: 1.81–5.03), Tai Chi (MD = 4.38, 95% CI: 2.28–6.49), and Yijinjing groups (MD = 5.52, 95% CI: 3.26–7.78) (*p* = 0.32).

Overall, both published meta-analyses and our re-meta-analysis consistently indicate that TCEs can significantly increase LVEF in patients with CHF.

#### 3.7.4 BNP and NT-proBNP

Prior SRs/MAs [31, 33, 34, 35, 39, 40, 43, 44] generally indicate that TCEs (*e*.*g*., Tai Chi, Baduanjin, Liuzijue) are associated with reductions in BNP and NT-proBNP levels, although the reported effect sizes vary. Mei *et al*. [31] found Baduanjin reduced NT-proBNP (standardized mean difference (SMD) = 0.62, 95% CI: 0.31–0.93; *p* < 0.01). Meanwhile, Bao *et al*. [33] reported decreases in BNP (MD = –56.80 pg/mL; *p* < 0.001) and NT-proBNP (MD = –174.94 pg/mL; *p* < 0.05). Hui *et al*. [34] observed Tai Chi lowered BNP/NT-proBNP (SMD = –1.12, 95% CI: –1.70 to –0.54; *p* = 0.0002). Yao *et al*. [35] reported significant BNP reductions (MD = –59.77, 95% CI: –82.85 to –36.70; *p* < 0.00001). Ren *et al*. [39] (SMD = –1.08) and Gu *et al*. [40] (SMD = –1.01) demonstrated that Tai Chi reduced BNP levels. Li *et al*. [43] reported Tai Chi decreased NT-proBNP (MD = –12.14), and Wei *et al*. [44] found Tai Chi reduced BNP levels (MD = –10.75). Taylor-Piliae and Finley [36] and Chen *et al*. [37] reported modest but significant reductions in NT-proBNP. Conversely, Liao *et al*. [38] found no significant between-group difference in BNP levels, and Pan *et al*. [41] reported no significant change in NT-proBNP, likely due to the small sample size and heterogeneity.

Our re-meta-analysis of original trials corroborated these findings. For NT-proBNP (nine RCTs), the pooled MD was –45.68 pg/mL (95% CI: –76.33 to –15.04), with moderate heterogeneity (*p* = 0.01; I^2^ = 58%; Fig. 4B). Baduanjin (MD = –74.70, 95% CI: –108.77 to –40.63) had a more pronounced effect than Tai Chi (MD = –16.57, 95% CI: –27.12 to –6.03). For BNP (10 RCTs), the overall MD was –110.73 pg/mL (95% CI: –154.92 to –66.53), with high heterogeneity (*p* < 0.01; I^2^ = 99%; Fig. 4C). Baduanjin (MD = –193.91, 95% CI: –202.61 to –185.20) achieved a significantly better effect than Tai Chi (MD = –91.44, 95% CI: –149.10 to –33.77) and combined exercise (MD = –68.94, 95% CI: –94.56 to –43.32).

Despite variations in the reported effect magnitudes, both prior syntheses and our re-meta-analysis consistently demonstrate that TCEs, particularly Baduanjin, significantly reduce BNP and NT-proBNP concentrations in CHF patients.

#### 3.7.5 VO_2_max

Existing SRs/MAs present mixed results in assessing maximal oxygen consumption (VO_2_max). Only three studies [31, 32, 37] demonstrated that Tai Chi and Baduanjin significantly increased VO_2_max (intervention durations of 3 to 12 months). In contrast, other studies [33, 34, 35, 41, 43, 44] found no significant differences between the TCE and control groups.

Our re-meta-analysis, which pooled 12 RCTs, reported that TCEs induced a significant improvement in VO_2_max (random-effects MD = 1.67, 95% CI: 0.97–2.36; Fig. 4D), with high heterogeneity (*p* < 0.01; I^2^ = 87%). The subgroup analyses showed that Yijinjing (MD = 3.10, 95% CI: 2.08–4.12) had a higher effect than Baduanjin (MD = 1.72, 95% CI: 0.56–2.88) and Tai Chi (MD = 1.22, 95% CI: 0.79–1.65). However, because Yijinjing was reported in only one study, these results may be more uncertain and variable.

Overall, despite some inconsistencies in the literature, both published meta-analyses and our re-meta-analysis suggest that TCEs promote potential benefits for aerobic capacity.

### 3.8 Bias Assessment and Sensitivity Analyses

The Egger’s and funnel plot tests were used to detect publication bias. According to Egger’s test, the results of the MLHFQ, 6-MWT, BNP, LVEF, and VO_2_max analyses did not indicate significant publication bias, thereby increasing confidence in the results of these studies. Although there are insufficient studies to perform Egger’s test for NT-proBNP, the symmetry of the funnel plot suggests that significant publication bias is potentially absent. Nevertheless, these findings should still be interpreted with caution and in conjunction with other methods or complementary studies for further affirmation (**Supplementary Table 8**).

To assess the robustness of the results, we also performed sensitivity analyses by excluding individual studies. The results showed that the overall effect and the reliability of the conclusions remained robust even after excluding any single study (**Supplementary Table 9**).

## 4. Discussion

In contrast to conventional cardiac rehabilitation exercises, TCEs stand out for their low intensity and minimal reliance on specific venues or schedules, making these exercises more accessible and easier to disseminate and implement. Moreover, the movements involved in these exercises are deliberately slow, gentle, steady, and varied. Furthermore, during practice, TCEs emphasise synchronising body movement with breathing and promote circulation through stretching, thereby relieving symptoms of heart failure and improving cardiopulmonary function. Furthermore, TCEs draw on TCM concepts such as “supporting Yang” and “nourishing Qi,” both of which aim to restore physiological balance.

A scientific statement by the American Heart Association [10] on “Complementary and Alternative Medicines in the Management of Heart Failure,” identified Tai Chi as a safe and well-tolerated adjunctive therapy for patients with heart failure and suggests that Tai Chi may be a beneficial form of exercise and cardiac rehabilitation. Cardiovascular benefits may arise from enhanced parasympathetic activity and reduced sympathetic drive [10]. This is thought to increase coronary collateral circulation, stroke volume, and cardiac output, thereby improving LVEF and alleviating symptoms in patients with heart failure [45, 46]. Exercises such as Baduanjin and Liuzijue also benefit the physical and mental health of patients with heart failure. For example, Baduanjin training in patients with ST-elevation myocardial infarction (STEMI) has been shown to mitigate adverse left ventricular remodelling by improving energy metabolism, suppressing inflammation, and modulating extracellular matrix organisation [47]. Consistent practice of Liuzijue can strengthen respiratory muscle endurance and enhance lung ventilation, potentially increasing activity tolerance in patients with heart failure [48, 49]. Additionally, Liuzijue can mitigate the inflammatory response by decreasing the levels of interleukin (IL)-4, IL-13, and IL-17 while simultaneously increasing IL-10 levels [50]. Yijinjing has also demonstrated potential benefits in heart failure rehabilitation; the stretching and rotational movements in Yijinjing appear to improve coronary blood supply and ease heart failure symptoms [51].

Our re-meta-analysis, which draws directly on data from 65 original RCTs, provides compelling evidence that TCEs, including Tai Chi, Baduanjin, Qigong, Liuzijue, and Yijinjing, benefit multiple clinical outcomes in patients with CHF. With intervention periods ranging from 1 to 12 months, these practices consistently increased 6-MWT distance, improved MLHFQ QoL scores, and raised LVEF; no material differences were detected among the exercise styles. TCEs also lowered BNP and NT-proBNP concentrations, with Baduanjin showing the most marked effect. Although all modalities were advantageous, Yijinjing appeared to yield the greatest gains in VO_2_max, a result that warrants confirmation in larger, well-designed trials. The magnitude of these effects on 6-MWT (+53 m), MLHFQ (-8.4 points), and LVEF (+4.1%) is broadly consistent with earlier syntheses by Pan *et al*. [41] and Gu *et al*. [40]; however, the increase in VO_2_max observed in our study (~1.7 mL⋅kg^-1^⋅min^-1^) exceeds previous estimates, probably because duplicate trials were removed and several recent RCTs were incorporated. These effect sizes align with and extend the signals noted in earlier reviews by Pan *et al*. [41] and Gu *et al*. [40], providing a more consolidated and up-to-date appraisal of the use of TCEs to treat CHF.

Recent high-quality studies lend additional support to the results of our re-meta-analysis. The multicenter RCT (the BEAR trial) evaluated Baduanjin as an adjunct to standard rehabilitation in patients with HFmrEF or HFpEF. Integrating a 12-week Baduanjin programme significantly improved 6-MWT (6.14% vs. 1.32% in controls), raised the anaerobic threshold (25.87% vs. 3.94%), and improved QoL (MLHFQ score fell by 16.8% vs. 3.99%), with no safety issues reported [15]. In addition, a contemporary network meta-analysis comparing several mind–body exercises in heart failure populations provided a detailed ranking of their relative effects. Tai Chi, Baduanjin, and yoga each improved LVEF, with Tai Chi performing best. Tai Chi was also the only modality that consistently lengthened 6-MWT, whereas pilates, dancing, and yoga were most effective for VO_2_max. For QoL, Liuzijue, Tai Chi, and Baduanjin all provided significant benefits, with Liuzijue showing the greatest advantage [16]. Collectively with our pooled estimates, these results suggest that the selection of a specific TCE modality could be tailored to individual rehabilitation goals (*e*.*g*., LVEF vs. exercise capacity), thereby enhancing precision in exercise prescriptions for CHF.

In addition to the physiological and functional improvements, TCEs confer meaningful mental health benefits, a factor critical for the comprehensive management of CHF. MAs indicate a reduction in anxiety and depressive symptoms across various chronic illnesses, including those with physical comorbidities [52]. Findings in other chronic conditions further support this broad applicability; for instance, a recent meta-analysis in cancer survivors reported that Tai Chi and Qigong interventions produce small-to-moderate improvements in both physical and mental health compared to passive controls, underscoring their utility in managing chronic illness-related distress beyond heart failure [53]. The psychological benefits extend to immediate effects as well; even short, acute sessions of Tai Chi have been shown to temporarily improve attention and significantly reduce perceived stress in middle-aged adults, suggesting immediate psychological relief that could enhance initial patient engagement and adherence to exercise regimens [54]. Furthermore, these mind–body exercises have been shown to positively modulate heart rate variability (HRV), a key indicator of autonomic nervous system balance that is crucial for cardiovascular health, and to reduce perceived stress [55]. Specific improvements in depression and HRV parameters with Tai Chi in older adults have also been demonstrated, suggesting a direct link between TCEs and neurophysiological benefits [56]. These psychophysiological effects may act synergistically with hemodynamic and anti-inflammatory mechanisms, such as increased vagal tone, improved endothelial function, and downregulation of IL-6 and TNF-α, to produce the multidimensional benefits observed in our analysis.

## 5. Limitations

Despite the encouraging findings of our re-meta-analysis, several limitations should be acknowledged. First, as detailed in our quality assessments, the methodological quality of many included SRs and MAs was suboptimal, which may affect the reliability of the pooled estimates and raise concerns about bias and heterogeneity. Second, the high degree of overlap among primary studies across reviews (CCA = 15.71%) is an important concern, as it may inflate effect sizes and potentially distort meta-analytic conclusions. Third, TCEs such as Tai Chi, Baduanjin, and Yijinjing vary considerably in style, intensity, and duration, and this lack of standardization makes it difficult to attribute outcomes to specific protocols or formulate precise clinical recommendations. In addition, although the inclusion of numerous Chinese studies improved comprehensiveness, it may also introduce potential cultural bias in outcome reporting, particularly for subjective measures such as QoL. Finally, heterogeneity in intervention protocols and patient characteristics across primary studies also contributed to variability in the results. Although our re-meta-analysis sought to mitigate some of these issues by directly repooling the original data and confirming robustness through sensitivity analyses, further large-scale and rigorously designed studies are still needed to strengthen the evidence base.

## 6. Conclusions

This umbrella review supports the feasibility of incorporating TCEs as a form of complementary medicine for patients with CHF. Based on our re-analysis of existing evidence, TCEs appear to confer potential benefits in exercise capacity, QoL, and cardiovascular functional markers.

While the current evidence base, characterised by limitations in methodological quality, high study overlap, and low certainty of evidence, precludes drawing strong, definitive conclusions regarding widespread clinical efficacy, these exercises may still represent a valuable adjunctive intervention for certain CHF patients. This is particularly relevant for individuals who are unable to participate in higher-intensity exercise programs due to physical limitations or preference. Clinicians considering TCEs should apply these exercises cautiously, integrating them appropriately into a comprehensive, individualized rehabilitation program under suitable guidance and monitoring.

To provide robust guidance for clinical practice, high-quality RCTs with rigorous designs and sufficient sample sizes are needed to improve the assessment of the impact of TCEs. Additional research into the mechanisms of action and long-term follow-up studies will facilitate a more comprehensive understanding of the benefits and limitations of TCEs, thereby strengthening the scientific basis for their use in the comprehensive management of CHF and ultimately enhancing patient outcomes and QoL.

## Data Availability

The datasets used and analyzed during the current study, including data collection forms, extracted data, data used for analyses, and analytic code, are available from the corresponding author upon reasonable request.

## Abbreviations

6-MWT, six-minute walk test; AMSTAR 2, A MeaSurement Tool to Assess systematic Reviews 2; BNP, B-type natriuretic peptide; CCA, corrected covered area; CHF, chronic heart failure; CI, confidence interval; CT, conventional treatment; GRADE, Grading of Recommendations Assessment, Development, and Evaluation; HF, heart failure; HFmrEF, heart failure with mildly reduced ejection fraction; HFpEF, heart failure with preserved ejection fraction; HFrEF, heart failure with reduced ejection fraction; HRV, heart rate variability; IL, interleukin; LVEF, left ventricular ejection fraction; MD, mean difference; MLHFQ, Minnesota Living with Heart Failure Questionnaire; NT-proBNP, N-terminal pro-B-type natriuretic peptide; NYHA, New York Heart Association; PRISMA, Preferred Reporting Items for Systematic Reviews and Meta-Analyses; PROSPERO, International Prospective Register of Systematic Reviews; QoL, quality of life; RCT, randomized controlled trial; ROBIS, Risk of Bias in Systematic Reviews; SMD, standardized mean difference; SRs/MAs, systematic reviews and meta-analyses; STEMI, ST-elevation myocardial infarction; TCEs, traditional Chinese exercises; TCM, traditional Chinese medicine; VO_2_max, maximal oxygen consumption.

## Supplementary Material

Supplementary material associated with this article can be found, in the online version, at https://doi.org/10.31083/RCM46055.
