# Supplementary material for: The Efficacy of Traditional Chinese Exercises in Patients With Chronic Heart Failure: An Umbrella Review and Meta-Analysis
Source: Rev Cardiovasc Med. 2026 Mar 20;27(3):46055. doi: 10.31083/RCM46055 (PMC13036533; doi:10.31083/RCM46055)
Supplement: Supplementary file 1 [file 2153-8174-27-3-46055-s1.zip › Supplementary Table 1 - search strategies.pdf]

Pubmed

#	query	result
**Heart failure**		
#1	"Heart Failure"[MeSH Terms]	156023
#2	"heart failure"[Title/Abstract] OR "cardia* decompensation"[Title/Abstract] OR "cardia* failure"[Title/Abstract] OR "cardia* incompetence"[Title/Abstract] OR "cardia* insufficiency"[Title/Abstract] OR "heart decompensation"[Title/Abstract] OR "heart insufficiency"[Title/Abstract] OR "myocardial failure"[Title/Abstract] OR "myocardial insufficiency"[Title/Abstract]	252630
#3	#1 OR #2<br>"Heart Failure"[MeSH Terms] OR "Heart Failure"[Title/Abstract] OR "cardia* decompensation"[Title/Abstract] OR "cardia* failure"[Title/Abstract] OR "cardia* incompetence"[Title/Abstract] OR "cardia* insufficiency"[Title/Abstract] OR "heart decompensation"[Title/Abstract] OR "heart insufficiency"[Title/Abstract] OR "myocardial failure"[Title/Abstract] OR "myocardial insufficiency"[Title/Abstract]	286214
**Traditional Chinese Exercise**		
#4	"Tai Ji"[MeSH Terms] OR "Qigong"[MeSH Terms]	1849
#5	"Tai Ji"[Title/Abstract] OR "Qigong"[Title/Abstract] OR "Tai Chi"[Title/Abstract] OR "taichi"[Title/Abstract] OR "chi kung"[Title/Abstract] OR "chigung"[Title/Abstract] OR "qi gong"[Title/Abstract] OR "ba duan jin"[Title/Abstract] OR "baduanjin"[Title/Abstract] OR "Eight section brocade"[Title/Abstract] OR "wuqinxi"[Title/Abstract] OR "wu qin xi"[Title/Abstract] OR "liuzijue"[Title/Abstract] OR "liu zi jue"[Title/Abstract] OR "chinese exercise*"[Title/Abstract] OR "chinese fitness"[Title/Abstract]	3795
#6	#4 OR #5<br>"Tai Ji"[MeSH Terms] OR "Qigong"[MeSH Terms] OR "Tai Ji"[Title/Abstract] OR "Qigong"[Title/Abstract] OR "Tai Chi"[Title/Abstract] OR "taichi"[Title/Abstract] OR "chi kung"[Title/Abstract] OR "chigung"[Title/Abstract] OR "qi gong"[Title/Abstract] OR "ba duan jin"[Title/Abstract] OR "baduanjin"[Title/Abstract] OR "Eight section brocade"[Title/Abstract] OR "wuqinxi"[Title/Abstract] OR "wu qin xi"[Title/Abstract] OR "liuzijue"[Title/Abstract] OR "liu zi jue"[Title/Abstract] OR "six character* formula"[Title/Abstract] OR "chinese exercise*"[Title/Abstract] OR "chinese fitness"[Title/Abstract]	3955
**Meta-Analysis**		
#7	"meta analysis"[Publication Type]	207414
#8	"meta analysis"[Title/Abstract] OR "data pooling*"[Title/Abstract] OR "systematic review*"[Title/Abstract] OR "meta	485678

	analyses"[Title/Abstract]	
#9	"meta analysis"[Publication Type] OR "meta analysis"[Title/Abstract] OR "data pooling*"[Title/Abstract] OR "systematic review*"[Title/Abstract] OR "meta analyses"[Title/Abstract]	505903
#10	#3 AND #6 AND #9<br>("Heart Failure"[MeSH Terms] OR ("Heart Failure"[Title/Abstract] OR "cardia* decompensation"[Title/Abstract] OR "cardia* failure"[Title/Abstract] OR "cardia* incompetence"[Title/Abstract] OR "cardia* insufficiency"[Title/Abstract] OR "heart decompensation"[Title/Abstract] OR "heart insufficiency"[Title/Abstract] OR "myocardial failure"[Title/Abstract] OR "myocardial insufficiency"[Title/Abstract])) AND ("Tai Ji"[MeSH Terms] OR "Qigong"[MeSH Terms] OR ("Tai Ji"[Title/Abstract] OR "Qigong"[Title/Abstract] OR "Tai Chi"[Title/Abstract] OR "taichi"[Title/Abstract] OR "chi kung"[Title/Abstract] OR "chigung"[Title/Abstract] OR "qi gong"[Title/Abstract] OR "ba duan jin"[Title/Abstract] OR "baduanjin"[Title/Abstract] OR "Eight section brocade"[Title/Abstract] OR "wuqinxi"[Title/Abstract] OR "wu qin xi"[Title/Abstract] OR "liuzijue"[Title/Abstract] OR "liu zi jue"[Title/Abstract] OR "six character* formula"[Title/Abstract] OR "chinese exercise*"[Title/Abstract] OR "chinese fitness"[Title/Abstract])) AND ("meta analysis"[Publication Type] OR ("meta analysis"[Title/Abstract] OR "data pooling*"[Title/Abstract] OR "systematic review*"[Title/Abstract] OR "meta analyses"[Title/Abstract]))	37

Web of science

#	query	result
**heart failure**		
#1	TS=("heart failure")	342470
#2	(((((((((((((((((((((TI=("heart failure")) OR AB=("heart failure")) OR TI=("cardia* decompensation")) OR AB=("cardia* decompensation")) OR TI=("cardia* failure")) OR AB=("cardia* failure")) OR TI=("cardia* incompetence")) OR AB=("cardia* incompetence")) OR TI=("cardia* insufficiency")) OR AB=("cardia* insufficiency")) OR TI=("cardia* insufficiency")) OR AB=("cardia* insufficiency")) OR TI=("heart incompetence")) OR AB=("heart incompetence")) OR TI=("heart insufficiency")) OR AB=("heart insufficiency")) OR TI=("insufficientia cardis")) OR AB=("insufficientia cardis")) OR TI=("myocardial failure")) OR AB=("myocardial failure")) OR TI=("myocardial insufficiency")) OR AB=("myocardial insufficiency")	273084
#3	#1 OR #2	353754
**Traditional Chinese Exercise**		
#4	(((TS=("tai ji")) OR TS=("qigong")) OR TI=("tai ji")) OR AB=("tai ji")) OR TI=("Taiji*"))	6069

	OR AB=("Taiji*")) OR TI=("Tai Chi")) OR AB=("Tai Chi")) OR TI=("taichi")) OR AB=("taichi")) OR TI=("chi kung")) OR AB=("chi kung")) OR TI=("chigung")) OR AB=("chigung")) OR TI=("qi gong")) OR AB=("qi gong")) OR TI=("qigong")) OR AB=("qigong")) OR TI=("ba duan jin")) OR AB=("ba duan jin")) OR TI=("baduanjin")) OR AB=("baduanjin")) OR TI=("Eight section brocade")) OR AB=("Eight section brocade")) OR TI=("wuqinxi")) OR AB=("wuqinxi")) OR TI=("wu qin xi")) OR AB=("wu qin xi")) OR TI=("five animal exercise*")) OR AB=("five animal exercise*")) OR TI=("liuzijue")) OR AB=("liuzijue")) OR TI=("liu zi jue")) OR AB=("liu zi jue")) OR TI=("liu zi jue")) OR AB=("liu zi jue")) OR TI=("chinese exercise*")) OR AB=("chinese exercise*")) OR TI=("chinese fitness")) OR AB=("chinese fitness")	
**Meta-Analysis**		
#5	(((((((((TS=("meta analysis")) OR TI=("meta analysis")) OR AB=("meta analysis")) OR TI=("Clinical Trial Overview*")) OR AB=("Clinical Trial Overview*")) OR TI=("Clinical Trial Overview*")) OR AB=("Clinical Trial Overview*")) OR TI=("Systematic review*")) OR AB=("Systematic review*")) OR TI=("Systematic review*")) OR AB=("Systematic review*")	564803
#6	#3 AND #4 AND #5	59

Cohrance

#	query	result
**heart failure**		14690
#1	MeSH descriptor: [Heart Failure] explode all trees	52935
#2	(‘heart failure' OR 'cardia* decompensation' OR 'cardia* failure' OR 'cardia* incompetence' OR 'cardia* insufficiency' OR 'heart decompensation' OR 'heart incompetence' OR 'heart insufficiency' OR 'insufficientia cardis' OR 'myocardial failure' OR 'myocardial insufficiency'):ti,kw,ab	
#3	#1 OR #2	52960
**Traditional Chinese Exercise**		
#4	MeSH descriptor: [Tai Ji] explode all trees	591
#5	MeSH descriptor: [Qigong] explode all trees	160
#6	('Taiji*' OR 'Tai Ji' OR 'Tai Chi' OR 'taichi' OR 'chi kung' OR 'chigung' OR 'qi gong' OR 'qigong' OR 'ba duan jin' OR 'baduanjin' OR 'Eight section brocade' OR 'wuqinxi' OR 'wu qin xi' OR 'five animal exercise*' OR 'liuzijue' OR 'liu zi jue' OR 'Six character* formula' OR 'chinese exercise*' OR 'chinese fitness'):ti,kw,ab	5304
#7	#4 OR #5 OR #6	

**Meta-Analysis**		
#8	MeSH descriptor: [Meta-Analysis as Topic] explode all trees	1626
#9	('Meta analysis' OR 'Clinical Trial Overview*' OR 'Data Pooling*' OR 'Systematic review*' OR 'meta analyses'):ti,kw,ab	33299
#10	#8 OR #9	33299
#11	#3 AND #7 AND #10	17

EMBASE

#	query	result
**heart failure**		
#1	'heart failure'/exp OR 'heart failure'	78639
#2	'heart failure':ab,kw,ti OR 'cardia* decompensation':ab,kw,ti OR 'cardia* failure':ab,kw,ti OR 'cardia* incompetence':ab,kw,ti OR 'cardia* insufficiency':ab,kw,ti OR 'heart decompensation':ab,kw,ti OR 'heart insufficiency':ab,kw,ti OR 'myocardial failure':ab,kw,ti OR 'myocardial insufficiency':ab,kw,ti	417001
#3	#1 OR #2	794551
**Traditional Chinese Exercise**		
#4	'tai chi'/exp OR 'tai chi'	5040
#5	'tai ji':ab,kw,ti OR 'qigong':ab,kw,ti OR 'tai chi':ab,kw,ti OR 'taichi':ab,kw,ti OR 'chi kung':ab,kw,ti OR 'chigung':ab,kw,ti OR 'qi gong':ab,kw,ti OR 'ba duan jin':ab,kw,ti OR 'baduanjin':ab,kw,ti OR 'eight section brocade':ab,kw,ti OR 'wuqinxi':ab,kw,ti OR 'wu qin xi':ab,kw,ti OR 'liuzijue':ab,kw,ti OR 'liu zi jue':ab,kw,ti OR 'chinese exercise*':ab,kw,ti OR 'chinese fitness':ab,kw,ti	5218
#6	#4 OR #5	6623
**Meta-Analysis**		
#7	'meta analysis (topic)'/exp OR 'meta analysis (topic)'	56817
#8	'meta analysis':ab,kw,ti OR 'data pooling*':ab,kw,ti OR 'systematic review*':ab,kw,ti OR 'meta analyses':ab,kw,ti	598400
#9	#7 OR #8	633370
#10	#3 AND #6 AND #9	77

CNKI 中国知网

#	query	result
1#	(SU=心力衰竭 + (TKA=心力衰竭 + 心脏衰竭)) AND (TKA=太极 + 八段锦 + 气功 + 六字诀 + 传统运动) AND	7

	(TKA=meta 分析 + 系统评价)	

WANFANG 万方

#	query	result
1#	主题:(心力衰竭) and 题名或关键词:(太极 or 八段锦 or 气功 or 六字诀 or 传统运动) and 题名或关键词:(meta 分析 or 系统评价)	12

VIP 维普

#	query	result
1#	M=(心力衰竭) and M=(太极 or 八段锦 or 气功 or 六字诀 or 传统运动) and M=(meta 分析 or 系统评价)	8

SinoMed

#	query	result
1#	"心力衰竭"[常用字段:智能] AND("太极"[常用字段:智能] OR "八段锦"[常用字段:智能] OR "气功"[常用字段:智能] OR "六字诀"[常用字段:智能] OR "传统运动"[常用字段:智能]) AND("meta 分析"[常用字段:智能] OR "系统评价"[常用字段:智能])	9

Other sources

(NCT01294111) OR (Tai Chi Training for Elderly People With Chronic Heart Failure)<br>(NCT01625819) OR (Exploring Behavioral Interventions to Improve Heart Failure)<br>(NCT00110227) OR (Tai Chi Mind-Body Therapy for Chronic Heart Failure)<br>(NCT04445753) OR (Tai Chi Exercise in Patients with Heart Failure)<br>(NCT02722213) OR (Mindfulness & Stress Management Study for Cardiac Patients)<br>(NCT04981197) OR (Effects of Baduanjin Exercise 0on Heart Failure Patients)<br>(NCT03229681) OR (Baduanjin Exercise for Patients With Chronic Heart Failure on Phase II Cardiac Rehabilitation)<br>(NCT03180320) OR (BESMILE-HF Study)<br>(NCT06521281) OR (The Effect of Chan-Chuang Qigong on Fatigue, Exercise Capacity and Quality of Lifpatients With Heart Failure)	9
