# Supplementary material for: The Efficacy of Traditional Chinese Exercises in Patients With Chronic Heart Failure: An Umbrella Review and Meta-Analysis
Source: Rev Cardiovasc Med. 2026 Mar 20;27(3):46055. doi: 10.31083/RCM46055 (PMC13036533; doi:10.31083/RCM46055)
Supplement: Supplementary file 1 [file 2153-8174-27-3-46055-s1.zip › Supplementary Table 2 - list of excluded studies.pdf]

Supplementary Table 2: Detailed information on excluded literature.

No.	Title	First Author	Year	Reasons for exclusion
1	Exercise-based cardiac rehabilitation for adults with atrial fibrillation	B.J.R.Buckley	2024	The title and abstract were not consistent with our research topic.
2	Effects of non-drug interventions on anxiety and depression in patients with heart failure: A systematic review based on bayesian network meta-analysis	Z.Zhao	2023	The title and abstract were not consistent with our research topic.
3	Effects of traditional mind-body movement therapy on chronic cardiopulmonary dyspnoea: a systematic review and meta-analysis	T.G.Zhang	2023	The title and abstract were not consistent with our research topic.
4	Efficacy of traditional Chinese exercises in improving anthropometric and biochemical indicators in overweight and obese subjects: A systematic review and meta-analysis	Q.Yang	2023	The title and abstract were not consistent with our research topic.
5	The western and Chinese exercise training for blood pressure reduction among hypertensive patients: An overview of systematic reviews	K.Tsoi	2023	The title and abstract were not consistent with our research topic.
6	Benefits, Facilitators, and Barriers of Alternative Models of Cardiac Rehabilitation-A QUALITATIVE SYSTEMATIC REVIEW	K.R.N.Platz	2023	The title and abstract were not consistent with our research topic.
7	Patients with heart failure describe benefits, facilitators and barriers to home and community based models of cardiac rehabilitation- what makes me move	K. R.N.Platz	2023	The title and abstract were not consistent with our research topic.
8	Effect of vinyasa yoga on frailty and anthropometric measurements in elderly individuals	M.Adaiçi	2023	The title and abstract were not consistent with our research topic.
9	Efficacy of baduanjin for treatment of fatigue: A systematic review and meta-analysis of randomized controlled trials	H.Liu	2023	The title and abstract were not consistent with our research topic.
10	Efficacy and safety of different traditional Chinese health exercises in patients with coronary heart disease combined with chronic heart failure: A network meta-analysis	X.W.Huang	2023	The title and abstract were not consistent with our research topic.
11	Clinical Outcomes Following Exercise Rehabilitation in People with Multimorbidity: A Systematic Review	K.Barker	2023	The title and abstract were not consistent with our research topic.
12	Effects of different exercise types on quality of life for patients with atrial fibrillation: A systematic review and meta-analysis	A.AbuElkhair	2023	The title and abstract were not consistent with our research topic.
13	Scoping review of transformative interventions, using validated quality of life assessment	H.Addington	2023	The title and abstract were not consistent with our research topic.

	tools in heart failure (hf) patients with depression			
14	Determining the safety and effectiveness of tai chi: A critical overview of 210 systematic reviews of controlled clinical trials	G.Y.Yang	2023	The title and abstract were not consistent with our research topic.
15	Updated meta-analysis assessing effects of baduanjin on cardiopulmonary functions of patients with coronary heart disease	D.Wang	2023	The title and abstract were not consistent with our research topic.
16	Does tai chi improve psychological well-being and quality of life in patients with cardiovascular disease and/or cardiovascular risk factors? A systematic review	G.Yang	2022	The title and abstract were not consistent with our research topic.
17	Traditional Chinese exercise for chronic diseases	X.Q.Wang	2022	The title and abstract were not consistent with our research topic.
18	Updated meta-analysis assessing effects of baduanjin on cardiopulmonary functions of patients with coronary heart disease	D.Wang	2022	The title and abstract were not consistent with our research topic.
19	Tai chi and qigong for trauma exposed populations: A systematic review	L.Barbara	2022	The title and abstract were not consistent with our research topic.
20	Effectiveness of Tai Chi on older adults: A systematic review of systematic reviews with re-meta-analysis	Y.L.Leona	2022	The title and abstract were not consistent with our research topic.
21	The effects of nonconventional exercise on functional capacity and quality of life in patients with heart failure: A systematic review	K.Hong	2022	The title and abstract were not consistent with our research topic.
22	Scoping review of interventions to improve quality of life in heart failure (HF) patients with depression in a clinical setting	H.Addington	2022	The title and abstract were not consistent with our research topic.
23	Tai chi for chronic illness management: synthesizing current evidence from meta-analyses of randomized controlled trials	L.Zhou	2021	The title and abstract were not consistent with our research topic.
24	Current status of cardiac rehabilitation in argentina	C.Zeballos	2021	The title and abstract were not consistent with our research topic.
25	Determining the safety and effectiveness of tai chi: A critical overview of 210 systematic reviews	G.Y.Yang	2021	The title and abstract were not consistent with our research topic.
26	Tai Chi for health and well-being: A bibliometric analysis of published clinical studies between 2010 and 2020	G.Y.Yang	2021	The title and abstract were not consistent with our research topic.
27	Effect of traditional asian exercise on patients with chronic heart failure: a protocol for network meta-analysis of randomised controlled trials	J.Xu	2021	The title and abstract were not consistent with our research topic.

28	Non-pharmacological management of hypertension	V.Narsingh	2021	The title and abstract were not consistent with our research topic.
29	A mixed comparisons of different intensities and types of physical exercise in patients with diseases related to oxidative stress: A systematic review and network meta-analysis	Z.Lu	2021	The title and abstract were not consistent with our research topic.
30	TaiChi and Qigong for Depressive Symptoms in Patients with Chronic Heart Failure: A Systematic Review with Meta-Analysis	W.Jiang	2021	The title and abstract were not consistent with our research topic.
31	Clinical evidence of tai chi exercise prescriptions: A systematic review	J.Huang	2021	The title and abstract were not consistent with our research topic.
32	Tai chi interventions for adults with cardiovascular disease: Insights from a comprehensive systematic review and meta-analysis	E.Gathright	2021	The title and abstract were not consistent with our research topic.
33	Tai Chi for Improving Chronic Primary Musculoskeletal Pain: Protocol for a Systematic Review	R.Gao	2021	The title and abstract were not consistent with our research topic.
34	Mind-body therapies from traditional Chinese medicine: Evidence map	Z.Lissandra	2021	The title and abstract were not consistent with our research topic.
35	Effectiveness of tai chi for health promotion for adults with health conditions: a scoping review of meta-analyses	K.Easwaran	2021	The title and abstract were not consistent with our research topic.
36	Tai Chi for improving balance and reducing falls: An overview of 14 systematic reviews	D.L.Zhong	2020	The title and abstract were not consistent with our research topic.
37	Effectiveness of tai chi exercise on overall quality of life and its physical and psychological components among older adults: A systematic review and meta analysis	E.Wang	2020	The title and abstract were not consistent with our research topic.
38	Effect of Tai Chi Chuan in Breast Cancer Patients: A Systematic Review and Meta-Analysis	X.C.Luo	2020	The title and abstract were not consistent with our research topic.
39	Effectiveness of tai chi on quality of life, depressive symptoms and physical function among community-dwelling older adults with chronic disease: A systematic review and meta-analysis	Y.T.Choo	2020	The title and abstract were not consistent with our research topic.
40	Benefits and harms of exercise therapy in people with multimorbidity: A systematic review and meta-analysis of randomised controlled trials	A.Bricca	2020	The title and abstract were not consistent with our research topic.
41	Effectiveness and safety of baduanjin exercise (BDJE) on heart failure with preserved left ventricular ejection fraction (HFpEF): a protocol for systematic review and meta-analysis	M.Cheng	2020	The title and abstract were not consistent with our research topic.
42	Benefits and harms of exercise therapy in people with multimorbidity: a systematic review	A.Bricca	2020	The title and abstract were not consistent with our research topic.

	and meta-analysis of randomised controlled trials			
43	Respiratory training interventions improve health status of heart failure patients: A systematic review and network meta-analysis of randomized controlled trials	M.H.Wang	2019	The title and abstract were not consistent with our research topic.
44	Depression in somatic disorders: Is there a beneficial effect of exercise?	A.Roeh	2019	The title and abstract were not consistent with our research topic.
45	The effects of tai chi mind-body approach on the mechanisms of gulf war illness: An umbrella review	K.F.Reid	2019	The title and abstract were not consistent with our research topic.
46	Six weeks of oral eChinacea purpurea supplementation does not enhance the production of serum erythropoietin or erythropoietic status in recreationally active males with above-average aerobic fitness	T.D.Martin	2019	The title and abstract were not consistent with our research topic.
47	The safety of tai chi: A meta-analysis of adverse events in randomized controlled trials	H.Cui	2019	The title and abstract were not consistent with our research topic.
48	The development and use of the assessment of dementia awareness and person-centred care training tool in long-term care	B.Creese	2019	The title and abstract were not consistent with our research topic.
49	Effects of tai chi on self-efficacy: A systematic review	Y. Tao	2018	The title and abstract were not consistent with our research topic.
50	Women's participation in stress management randomized controlled trials for chronic heart failure patients	L.A.J.Scott-Sheldon	2018	The title and abstract were not consistent with our research topic.
51	Reported methods for handling missing change standard deviations in meta-analyses of exercise therapy interventions in patients with heart failure: A systematic review	M.J.Pearson	2018	The title and abstract were not consistent with our research topic.
52	Lifestyle Therapy for the Management of Atrial Fibrillation	A.A.Abdul-Aziz	2018	The title and abstract were not consistent with our research topic.
53	Tai Chi Exercise for the Quality of Life in a Perimenopausal Women Organization: A Systematic Review	Z.Wang	2017	The title and abstract were not consistent with our research topic.
54	What to say if your patients ask about tai chi: evidence-based response	P.Huston	2017	The title and abstract were not consistent with our research topic.
55	CAMSTRAND conference 2016 abstracts	None	2016	The title and abstract were not consistent with our research topic.
56	Update in hypertension therapy	L.A.Mankin	2016	The title and abstract were not consistent with our research topic.
57	The effects of traditional Chinese exercise in patients with chronic obstructive pulmonary disease: A meta-analysis	K.T.Luo	2016	The title and abstract were not consistent with our research topic.
58	Complementary/integrative therapies that work: A review of the evidence	B.Kligler	2016	The title and abstract were not consistent with our research topic.

59	Patient engagement in randomized controlled tai chi clinical trials among the chronically ill	D.Jiang	2016	The title and abstract were not consistent with our research topic.
60	Health benefits of tai chi: what is the evidence?	P.Huston	2016	The title and abstract were not consistent with our research topic.
61	Health benefits of traditional Chinese sports and physical activity for older adults: A systematic review of evidence	Y.Guo	2016	The title and abstract were not consistent with our research topic.
62	Tai chi chuan for the primary prevention of stroke in middle-aged and elderly adults: A systematic review	F.Zheng	2015	The title and abstract were not consistent with our research topic.
63	Evidence base of clinical studies on tai chi: A bibliometric analysis	G.Y.Yang	2015	The title and abstract were not consistent with our research topic.
64	Effect of traditional Chinese exercise on the quality of life and depression for chronic diseases: A meta-analysis of randomised trials	L.Q.Wang	2015	The title and abstract were not consistent with our research topic.
65	Meditative Movement Therapies and Health-Related Quality-of-Life in Adults: A Systematic Review of Meta-Analyses	A.George	2015	The title and abstract were not consistent with our research topic.
66	The effect of Tai Chi and Qigong practice on depression and anxiety symptoms: A systematic review and meta-regression analysis of randomized controlled trials	J.C.Yin	2014	The title and abstract were not consistent with our research topic.
67	What do we really know about the safety of tai chi?: A systematic review of adverse event reports in randomized trials	M.Peter	2014	The title and abstract were not consistent with our research topic.
68	Effects of tai chi on health related quality of life in patients with chronic conditions: a systematic review of randomized controlled trials	YW.Chen	2014	The title and abstract were not consistent with our research topic.
69	So much research, so little application: Barriers to dissemination and practical implementation of tai ji quan	A.Peter	2014	The title and abstract were not consistent with our research topic.
70	Tai chi chuan in medicine and health promotion	C.Lan	2013	The title and abstract were not consistent with our research topic.
71	A review focused on the psychological effectiveness of tai chi on different populations	K.Zhang	2012	The title and abstract were not consistent with our research topic.
72	Effects of interventions on depression in heart failure: A systematic review	C.Patricia	2012	The title and abstract were not consistent with our research topic.
73	Tai Chi Exercise for Patients With Heart Disease: A Systematic Review of Controlled Clinical Trials	S.M.Ng	2012	The title and abstract were not consistent with our research topic.
74	Meta-analysis of the effect of cardiac rehabilitation interventions on depression outcomes in adults 64 years of age and older	Z.D.Gellis	2012	The title and abstract were not consistent with our research topic.

75	A systematic review of the effectiveness of qigong exercise in cardiac rehabilitation	C.Chan	2012	The title and abstract were not consistent with our research topic.
76	Quality of Reporting of Randomized Clinical Trials in Tai Chi Interventions-A Systematic Review	J.Y.Li	2011	The title and abstract were not consistent with our research topic.
77	Other complementary therapies	None	2010	The title and abstract were not consistent with our research topic.
78	Tai chi exercise for patients with cardiovascular conditions and risk factors: A SYSTEMATIC REVIEW	G.Y.Yeh	2009	The title and abstract were not consistent with our research topic.
79	The Effect of Tai Chi on Psychosocial Well-being: A Systematic Review of Randomized Controlled Trials	W.C.Wang	2009	The title and abstract were not consistent with our research topic.
80	Evidence from the cochrane collaboration for traditional Chinese medicine therapies	E.Manheimer	2019	The title and abstract were not consistent with our research topic.
81	Tai chi for cardiovascular disease and its risk factors: A systematic review	M.S.Lee	2007	The title and abstract were not consistent with our research topic.
82	(NCT04445753) OR (Tai Chi Exercise in Patients with Heart Failure)	Abant Izzet Baysal University	2024	The title and abstract were not consistent with our research topic.
83	(NCT06521281) OR (The Effect of Chan-Chuang Qigong on Fatigue, Exercise Capacity and Quality of Lifpatients With Heart Failure)	C.H.Hsieh	2024	The title and abstract were not consistent with our research topic.
84	(NCT04981197) OR (Effects of Baduanjin Exercise 0on Heart Failure Patients)	National Yang Ming Chiao Tung University	2022	The title and abstract were not consistent with our research topic.
85	(NCT03180320) OR (BESMILE-HF Study)	W.H.Lu	2021	The title and abstract were not consistent with our research topic.
86	(NCT02722213) OR (Mindfulness & Stress Management Study for Cardiac Patients)	University of Minnesota	2019	The title and abstract were not consistent with our research topic.
87	(NCT01294111) OR (Tai Chi Training for Elderly People With Chronic Heart Failure)	L.Hagglund	2018	The title and abstract were not consistent with our research topic.
88	(NCT00110227) OR (Tai Chi Mind-Body Therapy for Chronic Heart Failure)	G.Y.Yeh	2017	The title and abstract were not consistent with our research topic.
89	(NCT03229681) OR (Baduanjin Exercise for Patients With Chronic Heart Failure on Phase II Cardiac Rehabilitation)	Xiyuan Hospital of China Academy of	2017	The title and abstract were not consistent with our research topic.

		Chinese Medical Sciences		
90	(NCT01625819) OR (Exploring Behavioral Interventions to Improve Heart Failure)	S.Laura	2016	The title and abstract were not consistent with our research topic.
91	Mind-Body Interventions for Individuals With Heart Failure: A Systematic Review of Randomized Trials	G.M. Zehra	2018	Lack of relevant outcome indicators.
92	The effect of Tai Chi on four chronic conditionscancer, osteoarthritis, heart failure and chronic obstructive pulmonary disease: a systematic review and meta-analyses	Y.W.Chen	2015	Lack of relevant outcome indicators.
93	Traditional Chinese Exercise for Cardiovascular Diseases: Systematic Review and Meta-Analysis of Randomized Controlled Trials	X.Q.Wang	2016	Lack of relevant outcome indicators.
94	Meta-Analysis of the Efficacy of Baduanjin Combined with Conventional Therapy in Patients with Chronic Heart Failure	Q.Z.Wu	2023	Lack of relevant outcome indicators.
95	Systematic evaluation and meta-analysis of Taijiquan on cardiopulmonary function and efficacy in patients with chronic heart failure	R.S.Wen	2023	Lack of relevant outcome indicators.
96	Meta-analysis of traditional Chinese exercise on cardiopulmonary function and exercise tolerance in patients with chronic heart failure	Z.H.Hui	2023	Lack of relevant outcome indicators.
97	Effect of Baduanjin Intervention on Rehabilitation for Patients with Chronic Heart Failure:A Meta Analysis	Y.X.Liu	2023	Lack of relevant outcome indicators.
98	Meta-analysis of the effect of Tai Chi exercise on elderly patients with heart failure	H.Y.Jing	2023	Lack of relevant outcome indicators.
99	Meta-analysis of therapeutic effect of Baduanjin exercise on patients with chronic heart failure	Q.F.Li	2022	Lack of relevant outcome indicators.
100	Effects of Baduanjin Exercise on the Quality of Life among Patients with Chronic Heart Failure: A Meta-Analysis	Y.Wang	2019	Y.Wang
