# Supplementary material for: The Efficacy of Traditional Chinese Exercises in Patients With Chronic Heart Failure: An Umbrella Review and Meta-Analysis
Source: Rev Cardiovasc Med. 2026 Mar 20;27(3):46055. doi: 10.31083/RCM46055 (PMC13036533; doi:10.31083/RCM46055)
Supplement: Supplementary file 1 [file 2153-8174-27-3-46055-s1.zip › Supplementary Table 4 - GRADE evidence quality assessment.pdf]

Table 3 Results of evidence quality.

				Certainty assessment					No. of patients						
Citation	Outcomes	No. Of trails	Design	Limitations	Inconsistency	Indirectness	Imprecision	Publication bias	Experimental	Control	Relative effect (95% CI)	P-value	Quality	I^2	P for heterogeneity
Yang WY, 2023 (CHN)	QoL (MLHFQ)	5	RCT	serious[a]	serious[b]	no	no	no	189	191	MD -8.25 (-13.62,-2.89)	0.03	⊕⊕◯◯ Low	96%	<0.00001
	Physical QoL	4	RCT	serious[a]	serious[b]	no	no	no	179	181	MD -2.83 (-3.76,-1.90)	<0.00001	⊕⊕◯◯ Low	63%	0.04
	Emotional QOL	4	RCT	serious[a]	serious[b]	no	no	no	179	181	MD -2.52 (-3.67,-1.37)	0.0006	⊕⊕◯◯ Low	83%	<0.0001
	General QOL	2	RCT	serious[a]	serious[b]	no	serious[c]	no	89	91	MD -2.61 (-5.17,-0.06)	0.05	⊕◯◯◯ Very low	84%	0.01
	6-MWT	5	RCT	serious[a]	serious[b]	no	no	no	194	194	MD 118.49 (52.57,184.41)	0.0004	⊕⊕◯◯ Low	98%	<0.00001
Mei BW, 2023 (CHN)	QoL (MLHFQ)	12	RCT	serious[a]	no	no	no	serious[d]	397	398	SMD 1.10 (0.81,1.39)	<0.001	⊕⊕◯◯ Low	74.4%	0.01
	NT-Pro BNP	9	RCT	serious[a]	no	no	no	no	309	307	SMD 0.62 (0.31,0.93)	<0.001	⊕⊕⊕◯ Moderate	75.2%	0.72
	LVEF	7	RCT	serious[a]	serious[b]	no	no	serious[d]	293	289	SMD 0.53 (0.18,0.88)	<0.001	⊕◯◯◯ Very low	76.6%	0.23
	VO$_2$max	4	RCT	serious[a]	serious[b]	no	serious[c]	serious[d]	163	163	SMD 0.92 (0.24,1.60)	<0.001	⊕◯◯◯ Very low	74.7%	<0.001
Dai MQ, 2023 (CHN)	LVEF	13	RCT	serious[a]	serious[b]	no	serious[c]	no	491	477	MD 2.60 (1.17,4.02)	0.0004	⊕◯◯◯ Very low	94%	<0.00001
	VO$_2$max	4	RCT	serious[a]	serious[b]	no	no	no	147	148	MD 2.14 (1.02,3.26)	<0.001	⊕⊕◯◯ Low	94%	<0.00001
	AT	4	RCT	serious[a]	serious[b]	no	serious[c]	no	126	125	MD 1.61 (1.06,2.16)	<0.001	⊕◯◯◯ Very low	83%	<0.001
	QoL (MLHFQ)	17	RCT	serious[a]	serious[b]	no	serious[c]	no	634	614	MD 5.52 (3.17,7.88)	<0.001	⊕◯◯◯ Very low	95%	<0.001
	6-MWT	12	RCT	serious[a]	serious[b]	no	serious[c]	no	479	469	MD 36.83 (29.11,44.56)	<0.001	⊕◯◯◯ Very low	86%	<0.001
Bao QY, 2023 (CHN)	6-MWT	29	RCT	serious[a]	serious[b]	no	serious[c]	no	1165	1105	MD 72.82 (53.49,92.16)	<0.00001	⊕◯◯◯ Very low	94%	<0.00001
	VO$_2$max	4	RCT	serious[a]	serious[b]	no	serious[c]	no	109	108	MD 1.28 -(0.72,3.27)	0.21	⊕◯◯◯ Very low	68%	0.02
	LVEF	21	RCT	serious[a]	serious[b]	no	serious[c]	no	886	817	MD 5.09 (2.93,7.24)	<0.00001	⊕◯◯◯ Very low	95%	<0.00001
	BNP	12	RCT	serious[a]	serious[b]	no	serious[c]	no	527	483	MD -56.8 (-78.26,-35.33)	<0.00001	⊕◯◯◯ Very low	97%	<0.00001
	NT-Pro BNP	9	RCT	serious[a]	serious[b]	no	serious[c]	no	315	302	MD -174.94 (-318.14, -31.73)	0.02	⊕◯◯◯ Very low	98%	<0.00001
	QoL (MLHFQ)	21	RCT	serious[a]	serious[b]	no	serious[c]	no	712	699	MD -9.21 (-10.99,-7.42)	<0.001	⊕◯◯◯ Very low	93%	<0.00001
Hui JQ, 2022 (CHN)	QoL (MLHFQ)	9	RCT	serious[a]	serious[b]	no	no	no	395	381	MD -8.51 (-10.32,-6.7)	<0.00001	⊕⊕◯◯ Low	89%	<0.00001
	6-MWT	13	RCT	serious[a]	serious[b]	no	no	no	559	505	MD 43.47 (33.38,54.10)	<0.00001	⊕⊕◯◯ Low	83%	<0.00001
	LVEF	11	RCT	serious[a]	serious[b]	no	no	no	538	483	MD 6.07 (3.44,8.70)	<0.00001	⊕◯◯◯ Very low	98%	<0.00001
	BNP/NT-Pro BNP	9	RCT	serious[a]	serious[b]	no	no	no	368	324	MD -1.12 (-1.70,-0.54)	0.04	⊕⊕◯◯ Low	91%	<0.00001
	HAMD	2	RCT	serious[a]	serious[b]	no	no	no	82	81	MD -2.89 (-4.87,-0.91)	0.004	⊕⊕◯◯ Low	87%	0.005
	PSQI	2	RCT	serious[a]	serious[b]	no	no	no	82	81	MD -2.25 (-3.88,-0.61)	0.007	⊕⊕◯◯ Low	78%	0.03
	VO$_2$max	2	RCT	serious[a]	no	no	serious[c]	no	23	23	MD 1.38 (-1.51,4.28)	0.81	⊕⊕◯◯ Low	0%	0.35
	TUGT	2	RCT	serious[a]	no	no	no	no	55	55	MD -1.34 (-2.50,-0.90)	0.02	⊕⊕⊕◯ Moderate	0%	0.62
	Heart failure hospitalization	3	RCT	serious[a]	no	no	no	no	120	117	RR 0.47 (0.25,0.99)	0.02	⊕⊕⊕◯ Moderate	0%	0.52
Yao F, 2021 (CHN)	6-MWT	7	RCT	serious[a]	serious[b]	no	no	serious[d]	276	275	MD 67.23 (54.55,81.91)	<0.00001	⊕⊕⊕◯ Moderate	0%	0.65
	QoL (MLHFQ)	4	RCT	serious[a]	no	no	serious[c]	no	102	103	MD -9.51 (-17.84,-1.18)	0.03	⊕◯◯◯ Very low	51%	0.11
	LEVF	2	RCT	serious[a]	serious[b]	no	serious[c]	no	58	58	MD1.38 (-0.38,5.84)	0.54	⊕⊕◯◯ Low	3%	0.31
	BNP	4	RCT	serious[a]	serious[b]	no	serious[c]	no	164	164	MD-59.77 (-82.58,-36.70)	0.34	⊕◯◯◯ Very low	11%	<0.00001
	VO$_2$max	3	RCT	serious[a]	serious[b]	no	serious[c]	no	69	68	MD-0.04 (-1.62,1.54)	0.96	⊕⊕◯◯ Low	0%	0.45
	Number of rehospitalisation	2	RCT	serious[a]	serious[b]	no	serious[c]	no	120	120	MD-0.83 (-0.98,-0.68)	<0.00001	⊕⊕◯◯ Low	0%	1.00
	Number of hospitalisations and costs	2	RCT	serious[a]	serious[b]	no	serious[c]	no	120	120	MD-1.60 (-1.98,-1.31)	<0.00001	⊕⊕◯◯ Low	0%	1.00
Ruth TP, 2020 (USA)	6-MWT	5	RCT	serious[a]	serious[b]	no	Serious[c]	no	135	134	Hedges g 0.353 (0.041, 0.664)	0.026	⊕⊕⊕◯ Moderate	32.72 %	--
	QoL (MLHFQ)	5	RCT	serious[a]	serious[b]	no	Serious[c]	no	135	134	Hedges g −0.671 (−0.864, −0.370)	0.000	⊕⊕⊕◯ Moderate	0%	--

	Depression	4	RCT	serious[a]	serious[b]	no	Serious[c]	no	32	33	Hedges g -0.627 (-0.913,-0.341)	0.000	⊕◯◯◯ Very low	0%	--
	BNP	4	RCT	serious[a]	serious[b]	no	Serious[c]	no	103	103	Hedges g −0.333 (−0.604, −0.062)	0.016	⊕◯◯◯ Very low	0%	--
Liao Y, 2020 (CHN)	VO₂max	5	RCT	serious[a]	serious[b]	no	Serious[c]	no	105	101	MD 1.24 (0.74, 1.74)	0.002	⊕◯◯◯ Very low	58%	0.04
	NT-proBNP	7	RCT	serious[a]	no	no	Serious[c]	Serious[d]	225	212	MD -68.21(-164.72,28.31)	0.17	⊕◯◯◯ Very low	42%	0.11
	6-MWT	16	RCT	serious[a]	serious[b]	no	Serious[c]	Serious[d]	546	541	MD 40.03 (32.52,47.53)	<0.0001	⊕◯◯◯ Very low	90%	<0.0001
	QoL (MLHFQ)	14	RCT	serious[a]	serious[b]	no	Serious[c]	Serious[d]	463	457	MD -5.41 (-8.13,-2.70)	<0.0001	⊕◯◯◯ Very low	97%	<0.0001
	LVEF	12	RCT	serious[a]	serious[b]	no	Serious[c]	Serious[d]	440	432	MD 4.63 (2.31,6.91)	<0.0001	⊕◯◯◯ Very low	100%	<0.0001
	BNP	6	RCT	serious[a]	serious[b]	no	Serious[c]	Serious[d]	203	203	MD 2.0 (-62.01,66.01)	0.95	⊕◯◯◯ Very low	100%	<0.0001
Chen XK, 2020 (CHN)	QoL (MLHFQ)	14	RCT	serious[a]	no	no	Serious[c]	Serious[d]	100		MD -8.63 (-10.60,-6.67)	--	⊕◯◯◯ Very low	94%	--
	6-MWT	17	RCT	serious[a]	no	no	Serious[c]	Serious[d]	1461		MD 56.52 (41.27,71.78)	--	⊕◯◯◯ Very low	88%	--
	VO₂max	4	RCT	serious[a]	no	no	Serious[c]	Serious[d]	245		MD 1.24 (0.91,1.57)	--	⊕◯◯◯ Very low	0%	--
	LVEF	11	RCT	serious[a]	no	no	Serious[c]	Serious[d]	868		MD 3.97 (1.22 , 6.72)	--	⊕◯◯◯ Very low	96%	--
	BNP	8	RCT	serious[a]	no	no	Serious[c]	Serious[d]	691		MD -76.12 (-134.61,-17.62)	--	⊕◯◯◯ Very low	95%	--
Wang AZ, 2020 (CHN)	QoL (MLHFQ)	6	RCT	serious[a]	no	no	no	no	207	208	MD -10.88 (-13.45,-8.22)	<0.0001	⊕⊕⊕◯ Moderate	99%	<0.0001
	6-MWT	6	RCT	serious[a]	serious[b]	no	no	Serious[d]	227	225	MD 107.81 (75.83,139.78)	<0.0001	⊕◯◯◯ Very low	99%	<0.0001
	LVEF	4	RCT	serious[a]	no	no	Serious[c]	Serious[d]	147	145	MD 3.62 (2.04,5.19)	<0.0001	⊕◯◯◯ Very low	8%	0.35
	NT-proBNP	2	RCT	serious[a]	serious[b]	no	Serious[c]	Serious[d]	87	85	MD -59.32 (-284.84,166.20)	0.61	⊕◯◯◯ Very low	0%	0.85
	BNP	2	RCT	serious[a]	serious[b]	no	Serious[c]	Serious[d]	60	60	MD 4.00 (1.35,11.83)	0.01	⊕◯◯◯ Very low	28%	0.24
Li JC, 2018 (CHN)	LVEF	3	RCT	serious[a]	serious[b]	no	Serious[c]	no	128	108	MD 9.01 (6.33,11.69)	<0.0001	⊕◯◯◯ Very low	71%	0.03
	6-MWT	5	RCT	serious[a]	no	no	Serious[c]	no	161	151	MD 0.85 (0.61,1.08)	<0.0001	⊕⊕◯◯ Low	29%	0.23
	VO₂max	2	RCT	serious[a]	serious[b]	no	Serious[c]	Serious[d]	23	23	MD 1.03 (-1.71,3.78)	0.46	⊕◯◯◯ Very low	0%	0.97
	QoL (MLHFQ)	4	RCT	serious[a]	serious[b]	no	Serious[c]	Serious[d]	131	122	MD -1.10 (-1.91,-0.29)	0.008	⊕◯◯◯ Very low	84%	0.003
	NT-proBNP	2	RCT	serious[a]	serious[b]	no	Serious[c]	Serious[d]	45	45	MD -12.14 (-23.78,-0.50)	0.04	⊕◯◯◯ Very low	0%	0.57
Wei HY, 2017 (CHN)	QoL (MLHFQ)	7	RCT	serious[a]	serious[b]	no	no	no	279	270	MD −9.37 (−13.09, −5.65)	<0.0001	⊕⊕◯◯ Low	76%	0.0004
	6-MWT	7	RCT	serious[a]	serious[b]	no	no	no	277	267	MD 40.37 (9.48, 71.27)	0.01	⊕⊕◯◯ Low	82%	<0.0001
	VO₂max	3	RCT	serious[a]	no	no	Serious[c]	Serious[d]	73	73	MD 0.29 (−1.23, 1.81)	0.71	⊕◯◯◯ Very low	0%	0.82
	LVEF	5	RCT	serious[a]	serious[b]	no	no	no	212	202	MD 7.89 (3.01, 12.77)	0.002	⊕⊕◯◯ Low	95%	<0.0001
	BNP	5	RCT	serious[a]	no	no	no	no	162	162	MD −10.75 (−13.20, −8.30)	<0.0001	⊕⊕⊕◯ Moderate	0%	0.96
	SBP	4	RCT	serious[a]	no	no	Serious[c]	Serious[d]	80	81	MD −2.81 (−8.52, 2.90)	0.33	⊕◯◯◯ Very low	0%	0.55
	DBP	3	RCT	serious[a]	no	no	Serious[c]	Serious[d]	70	71	MD 0.37 (−3.73, 4.48)	0.86	⊕◯◯◯ Very low	0%	0.86
Ren XM,2017 (CHN)	6-MWT	7	RCT	serious[a]	serious[b]	no	no	no	241	233	WMD 65.29 (−32.55, 98.04)	<0.001	⊕⊕◯◯ Low	93%	0.00
	QoL (MLHFQ)	7	RCT	serious[a]	serious[b]	no	no	no	236	230	WMD −11.52 (−16.5, −6.98)	<0.001	⊕⊕◯◯ Low	87%	0.00
	BNP	5	RCT	serious[a]	serious[b]	no	no	no	133	133	SMD −1.08 (−1.91, −0.26)	<0.001	⊕⊕◯◯ Low	88%	<0.0002
	LVEF	5	RCT	serious[a]	serious[b]	no	no	no	200	180	SMD −1.08 (−1.91, −0.26)	<0.001	⊕⊕◯◯ Low	98%	0.00
Gu Q, 2017 (CHN)	6-MWT	10	RCT	serious[a]	serious[b]	no	no	no	344	379	WMD 51.01 (30.49, 71.53)	<0.001	⊕⊕◯◯ Low	86%	<0.0001
	QoL (MLHFQ)	8	RCT	serious[a]	serious[b]	no	no	no	280	318	WMD −10.37 (−14.43, −6.32)	<0.001	⊕⊕◯◯ Low	97%	<0.0001
	LVEF	7	RCT	serious[a]	serious[b]	no	no	no	283	306	WMD 7.72% (3.58, 11.89)	0.003	⊕⊕◯◯ Low	96%	<0.0001
	BNP	6	RCT	serious[a]	serious[b]	no	no	no	178	221	SMD −1.01(−1.82, −0.19)	0.02	⊕⊕◯◯ Low	92%	<0.0001
Pan L, 2013 (CHN)	6-MWT	3	RCT	serious[a]	no	no	Serious[c]	no	95	595	MD 46.73 (−1.62, 95.09)	0.06	⊕◯◯◯ Very low	91%	<0.0001
	QoL (MLHFQ)	3	RCT	serious[a]	serious[b]	no	Serious[c]	no	90	92	WMD −14.54 (−23.45, −5.63)	0.001	⊕◯◯◯ Very low	71%	0.001
	NT-proBNP	2	RCT	serious[a]	serious[b]	no	Serious[c]	Serious[d]	45	45	MD −61.16 (−179.27, −56.95)	0.31	⊕◯◯◯ Very low	76%	0.04
	SBP	2	RCT	serious[a]	serious[b]	no	Serious[c]	Serious[d]	55	57	MD −1.06 (−13.76, 11.63)	0.87	⊕◯◯◯ Very low	91%	0.0008
	DBP	2	RCT	serious[a]	serious[b]	no	Serious[c]	Serious[d]	55	57	MD −0.08 (−3.88, 3.73)	0.97	⊕◯◯◯ Very low	56%	0.13
	VO₂max	2	RCT	serious[a]	no	no	Serious[c]	Serious[d]	65	65	MD 0.19 (−0.74, 1.13)	0.19	⊕◯◯◯ Very low	0%	0.45

Table Notes:

BNP, B-type natriuretic peptide; CI, confidence interval; HAMD, Hamilton Depression Rating Scale; LVEF, left ventricular ejection fraction; MD, mean difference; MLHFQ, Minnesota Living with Heart Failure Questionnaire; NT-pro-BNP, N-terminal fragment of pro-BNP; Peak VO2, peak oxygen uptake; PSQI, Pittsburgh Sleep Quality Index; RR, risk ratio; SMD, standardized mean difference; TUGT, timed up and go test; 6-MWT, 6-min walk test.

a.The experimental design had a large bias in random, distributive findings or was blind.

b.The confidence interval overlap less, the heterogeneity test p was very small, and the I2 was larger.

c.The Confidence interval was not narrow enough, or the simple size is too small.

d.Funnel graph asymmetry, or fewer studies were included and there may have been greater publication bias.

Legend for Symbols:

⊕⊕⊕⊕ High-quality evidence

⊕⊕⊕◯ Moderate-quality evidence

⊕⊕◯◯ Low-quality evidence

⊕◯◯◯ Critical Low-quality evidence
