# Supplementary material for: The Efficacy of Traditional Chinese Exercises in Patients With Chronic Heart Failure: An Umbrella Review and Meta-Analysis
Source: Rev Cardiovasc Med. 2026 Mar 20;27(3):46055. doi: 10.31083/RCM46055 (PMC13036533; doi:10.31083/RCM46055)
Supplement: Supplementary file 1 [file 2153-8174-27-3-46055-s1.zip › Supplementary Table 5 - overlapping of included studies.pdf]

Supplementary Table 3: Summary of the primary RCTs included in the 15 SRs/MAs and CCA calculation process.

Study ID	W.Y.Yang, 2023	B.W.Mei , 2023	M.Q.Dai , 2023	Q.Y.Bao, 2023	J.Q.Hui, 2022	F.Yao , 2021	R.Taylor-Piliae, 2021	Y.Liao, 2020	X.Chen, 2020	Ren XM,2017	A.T.Wang, 2020	J.C.Li, 2018	H.Y.Wei, 2017	Q.Gu, 2017	L.Pan, 2013
R.L.Li,2017	YES	YES	NO	YES	NO	NO	NO	NO	YES	NO	YES	NO	NO	NO	NO
X.Y.Li,2018	YES	YES	NO	NO	NO	YES	NO	YES	YES	NO	YES	NO	NO	NO	NO
D.M.Chen,2018	YES	YES	NO	YES	YES	NO	YES	NO	YES	NO	YES	NO	NO	NO	NO
H.Zhou,2019	YES	NO	NO	YES	NO	NO	NO	NO	NO	NO	NO	NO	NO	NO	NO
H.L.Lu,2019	YES	YES	NO	YES	NO	NO	NO	YES	NO	NO	NO	NO	NO	NO	NO
M.Xu,2019	YES	NO	NO	YES	NO	NO	NO	YES	NO	NO	NO	NO	NO	NO	NO
W.Qi,2020	YES	NO	YES	NO	NO	YES	NO	YES	NO	NO	NO	NO	NO	NO	NO
Y.J.Jiao,2020	YES	YES	NO	YES	NO	NO	NO	NO	NO	NO	NO	NO	NO	NO	NO
X.H.Xiong,2016	NO	YES	YES	YES	NO	NO	YES	NO	YES	NO	YES	NO	NO	NO	NO
S.Fang,2022	NO	YES	NO	NO	NO	NO	NO	NO	NO	NO	NO	NO	NO	NO	NO
H.M.Tang,2019	NO	YES	NO	NO	NO	NO	YES	NO	NO	NO	NO	NO	NO	NO	NO
L.Zhang,2022	NO	YES	YES	NO	NO	NO	NO	NO	NO	NO	NO	NO	NO	NO	NO
M.L.Yu,2018	NO	YES	NO	NO	NO	NO	YES	NO	YES	NO	YES	NO	NO	NO	NO
X.K.Hong,2020	NO	YES	NO	NO	NO	NO	NO	NO	NO	NO	NO	NO	NO	NO	NO
Y.Xu,2022	NO	YES	NO	NO	NO	NO	NO	NO	NO	NO	NO	NO	NO	NO	NO
W.Pan,2019	NO	YES	NO	YES	NO	NO	YES	NO	NO	NO	NO	NO	NO	NO	NO
Y.Wang,2021	NO	YES	NO	NO	NO	NO	NO	NO	NO	NO	NO	NO	NO	NO	NO
L.Ye,2021	NO	YES	NO	YES	NO	NO	NO	NO	NO	NO	NO	NO	NO	NO	NO

X.L.Chen,2021	NO	NO	YES	NO	NO	NO	NO	NO	NO	NO	NO	NO	NO	NO	NO
H.J.Li,2017	NO	NO	YES	NO	NO	NO	NO	NO	NO	NO	NO	NO	NO	NO	NO
C.D.Yao,2010	NO	NO	YES	YES	YES	NO	NO	NO	YES	YES	NO	YES	YES	YES	NO
Yang HX,2021	NO	NO	YES	YES	YES	NO	NO	NO	NO	NO	NO	NO	NO	NO	NO
Y.W.Ke,2021	NO	NO	YES	NO	NO	NO	NO	NO	NO	NO	NO	NO	NO	NO	NO
X.F.Deng,2019	NO	NO	YES	NO	NO	NO	NO	NO	NO	NO	NO	NO	NO	NO	NO
X.T.Wang,2022	NO	NO	YES	NO	NO	NO	NO	NO	NO	NO	NO	NO	NO	NO	NO
L.Zheng,2017	NO	NO	YES	NO	NO	NO	NO	NO	YES	NO	NO	NO	NO	NO	NO
W.J.Feng,2017(a)	NO	NO	YES	NO	NO	NO	NO	YES	NO	NO	NO	NO	NO	NO	NO
G.Caminiti,2011	NO	NO	NO	YES	YES	NO	YES	YES	YES	YES	YES	YES	NO	YES	YES
H.Zhou,2015	NO	NO	NO	YES	NO	NO	NO	NO	NO	NO	NO	NO	NO	NO	NO
G.Y.Yeh,2013	NO	NO	NO	YES	YES	YES	YES	YES	YES	YES	YES	YES	NO	YES	NO
B.Zhou,2020	NO	NO	NO	YES	YES	NO	NO	NO	NO	NO	NO	NO	NO	NO	NO
X.J.Yang,2015	NO	NO	NO	YES	NO	YES	NO	NO	YES	NO	NO	NO	NO	NO	NO
X.Yan,2016	NO	NO	NO	YES	NO	YES	NO	YES	NO	NO	NO	NO	NO	NO	NO
J.H.Ke,2020	NO	NO	NO	YES	NO	NO	NO	NO	NO	NO	NO	NO	NO	NO	NO
D.Wei,2003	NO	NO	NO	YES	NO	NO	NO	NO	NO	YES	YES	NO	NO	YES	NO
D.Yu,2019	NO	NO	NO	YES	NO	NO	NO	YES	YES	NO	NO	NO	NO	NO	NO
J.Yu,2015	NO	NO	NO	YES	NO	NO	NO	NO	YES	NO	NO	NO	NO	NO	NO
M.L.Yu,2020	NO	NO	NO	YES	YES	NO	NO	NO	NO	NO	NO	NO	NO	NO	NO
Z.H.Zhang,2021	NO	NO	NO	YES	NO	NO	NO	NO	NO	NO	NO	NO	NO	NO	NO
G.Y.Yeh,2004	NO	NO	NO	YES	YES	YES	YES	NO	YES	YES	YES	YES	NO	YES	YES
G.Y.Yeh,2008	NO	NO	NO	YES	NO	NO	NO	YES	NO	YES	NO	NO	NO	YES	NO
G.Y.Yeh,2011	NO	NO	NO	YES	NO	YES	YES	YES	YES	YES	NO	YES	NO	YES	YES
L.S.Redwine,2019	NO	NO	NO	YES	NO	NO	YES	YES	YES	NO	NO	NO	NO	NO	NO

L.Hägglund,2018	NO	NO	NO	YES	NO	NO	NO	YES	YES	NO	NO	NO	NO	NO	NO
T.Yu,2019	NO	NO	NO	YES	YES	NO	NO	YES	YES	NO	NO	NO	NO	NO	NO
L.M.Deng,2021	NO	NO	NO	YES	NO	NO	NO	NO	NO	NO	NO	NO	NO	NO	NO
Z.L.Kang,2021	NO	NO	NO	YES	NO	NO	NO	NO	NO	NO	NO	NO	NO	NO	NO
X.D.Deng,2018	NO	NO	NO	YES	NO	NO	NO	NO	YES	NO	NO	NO	NO	NO	NO
X.F.Pan,2016	NO	NO	NO	YES	YES	NO	NO	NO	YES	NO	NO	NO	NO	YES	NO
L.Sang,2015(a)	NO	NO	NO	YES	YES	NO	NO	YES	YES	YES	NO	NO	YES	YES	NO
Y.X.Zhou,2021	NO	NO	NO	YES	NO	NO	NO	NO	NO	NO	NO	NO	NO	NO	NO
L.Sang,2015(b)	NO	NO	NO	YES	YES	NO	NO	NO	NO	YES	NO	NO	YES	YES	NO
C.Li,2015	NO	NO	NO	YES	NO	YES	NO	NO	YES	NO	NO	NO	NO	NO	NO
L.H.Yuan,2016	NO	NO	NO	YES	NO	NO	NO	YES	YES	NO	NO	NO	NO	NO	NO
N.Wang,2011	NO	NO	NO	NO	YES	NO	NO	NO	YES	NO	NO	YES	NO	YES	NO
L.Yuan,2017	NO	NO	NO	NO	YES	NO	NO	NO	NO	NO	NO	NO	NO	NO	NO
F.Ding,2018	NO	NO	NO	NO	YES	NO	NO	NO	NO	NO	NO	NO	NO	NO	NO
D.E.Borrow,2007	NO	NO	NO	NO	NO	NO	YES	NO	YES	YES	NO	NO	YES	YES	YES
C.Huang,2014	NO	NO	NO	NO	NO	NO	NO	NO	YES	NO	NO	NO	YES	YES	NO
L.S.Redwine,2012	NO	NO	NO	NO	NO	NO	NO	NO	YES	NO	NO	NO	NO	NO	NO
Xiong XH,2017	NO	NO	NO	NO	NO	NO	NO	NO	YES	NO	NO	NO	NO	NO	NO
Z.Shi,2018	NO	NO	NO	NO	NO	NO	NO	NO	YES	NO	NO	NO	NO	NO	NO
R.Zheng,2018	NO	NO	NO	NO	NO	NO	NO	NO	YES	NO	NO	NO	NO	NO	NO
X.X.Li,2017	NO	NO	NO	NO	NO	NO	NO	NO	NO	NO	YES	NO	NO	NO	NO
S.Sato,2010	NO	NO	NO	NO	NO	NO	NO	NO	NO	NO	NO	NO	YES	NO	NO
**Total RCTs included**	8	15	21	41	15	9	6	22	33	7	7	10	11	13	4

Notes: SRs/MAs: systematic reviews and meta-analyses; RCTs: randomized controlled trials; CCA: corrected covered area. The tabular data are

presented in columns corresponding to a total of 65 unique primary RCTs and rows corresponding to the 15 SRs/MAs. “YES ” indicate when a primary RCT is included in an SR/MA. The total number of primary RCTs included in each SR/MA is presented in the last row. The total number of times a primary RCT is included in SRs/MAs is presented in the last columns. The total number of RCTs included in SRs/MAs is taken as "*N*" (repetition allowed), the total number of RCTs is "*r*" and the number of included SRs/MAs is "*c*", CCA= $(N - r) / [(r \times c) - r]$. $N = 208$, $r = 65$, $c = 15$.

List of the primary RCTs

NO.	Study ID	References
1	R.L.Li,2017	Observation on the improvement of cardiac function and quality of life in elderly patients with chronic heart failure by Baduanjin, J. Massage Rehabilitation Med. 8 (24) (2017) 23–25.
2	X.Y.Li,2018	The effect of Baduanjin combined with conventional western medicine therapy and health education on cardiac function in patients with heart failure with preserved ejection fraction, Int. J. Chinese Med. Materia Medica 40 (2018) 698–701, 08.
3	D.M.Chen,2018	The effects of Baduanjin exercise on fatigue and quality of life in patients with heart failure: a randomized controlled trial, Eur. J. Cardiovasc. Nurs. 17 (5) (2018).
4	H.Zhou,2019	The effect of baduanjin on cardiac rehabilitation in patients with heart failure, Guangming Journal Of Chinese Medicine 34 (14) (2019) 2248–2250.
5	H.L.Lu,2019	Effect of sitting-style Ba Duan Jin on quality of life in patients with heart function III~IV table heart failure, in: Cardiovascular Disease Journal of integrated traditional Chinese and Western Medicine, 7, 2019, pp. 1–2+4, 08.
6	M.Xu,2019	The effect of horizontal Baduanjin rehabilitation exercise on patients with stable heart failure of grade III-IV cardiac function impact on quality of life, Electr. J. Clinical Med. Literature 6 (99) (2019) 33–34.
7	W.Qi,2020	The effect of Baduanjin on cardiac function and quality of life in patients with chronic heart failure, Beijing Med. J. 42 (2020) 263–265, 03.
8	Y.J.Jiao,2020	Effects of Baduanjin on cardiac function and daily life in patients with chronic heart failure, J. Practical Trad. Chinese Med. 36 (12) (2020) 1635–1636.
9	X.H.Xiong,201	Therapeutic effect of Baduanjin on patients with coronary heart disease and chronic heart failure, China Mod. Med. J. 18 (2016) 55–56.

	6	
10	S.Fang,2022	Effect of Baduanjin on exercise tolerance in elderly patients with heart failure and frailty, Chin. J. Rehabil. Med. 37 (2022) 108–111.
11	H.M.Tang,2019	The Clinical Research on the Treatment of Chronic Heart Failure with Baduanjin Thearpy, Guangzhou Univ. Chinese Med., 2019, pp. 1–33
12	L.Zhang,2022	Clinical study on the effect of Baduanjin on heart function and psychology in patients with heart failure, Cont. Med. Educ. 36 (2022) 137–140.
13	M.L.Yu,2018	A randomized controlled study on the application of Baduanjin in patients with coronary heart disease and chronic heart failure, Beijing Univ. Tradit. Chinese Med. 5 (2018) 61–95.
14	X.K.Hong,2020	Clinical study of baduanjin in patients with chronic heart failure, Fujian Univ. Tradit. Chinese Med. (2020) 1–27.
15	Y.Xu,2022	Influence of Baduanjin exercise rehabilitation nursing on the rehabilitation of patients with chronic heart failure, China Mod. Med. 29 (2022) 157–163.
16	W.Pan,2019	Application effect of Baduanjin in cardiac rehabilitation of patients with chronic heart failure, GUANGMING J. CHINESE Med. 34 (2019) 3354–3356.
17	Y.Wang,2021	Effect of Baduanjin on cardiac rehabilitation in elderly patients with chronic heart failure, Chinese J. Gerontol. 41 (2021) 4260–4263.
18	L.Ye,2021	The effect of Baduanjin on cardiopulmonary function rehabilitation in patients with chronic heart failure. Zhejiang J. Tradit. Chin. Med. 2021, 56, 423.
19	X.L.Chen,2021	Feasibility and preliminary effects of the besmile-hf program on chronic heart failure patients: a pilot randomized controlled trial. Front Cardiovasc Med. (2021) 8:715207. doi: 10.3389/fcvm.2021.715207
20	H.J.Li,2017	Clinical efficacy of tai chi exercise in the treatment of chronic heart failure in coronary heart disease (MA thesis). Guangzhou University of Chinese Medicine (2017).
21	C.D.Yao,2010	The effect of tai chi exercises on the rehabilitation of patients with chronic heart failure. Chinese J Cardiovasc Rehabil Med. (2010) 19:364-7.
22	Yang HX,2021	Effects of Tai Chi rehabilitation exercise on cardiac function and blood lipid levels in elderly patients with congestive heart failure. Chinese J Cardiovasc Rehab Med. (2021) 30:382–7. doi: 10.3969/j.issn.1008-0074.2021.04.03
23	Y.W.Ke,2021	Clinical efficacy of Yiji Jing in patients with chronic heart failure (MA thesis). Fujian University of Chinese Medicine (2021).
24	X.F.Deng,2019	A clinical study of Baduan Jin on the rehabilitation of patients with chronic heart failure (MA thesis)

25	X.T.Wang,2022	Clinical efficacy of tai chi in patients with heart failure with preserved ejection fraction: a randomized controlled study. Chin J Integr Traditional Western. (2022) 42:961–7. doi: 10.7661/j.cjim.20210819.057
26	L.Zheng,2017	The effect of fitness qigong six words on heart function in patients with chronic heart failure. Chin J Evid Based Cardiovasc. (2017) 9:659–62. doi: 10.3969/j.issn.1674-4055.2017.06.05
27	W.J.Feng,2017(a)	Effects of Tai Chi on cardiopulmonary function in patients with chronic heart failure (MA thesis). Liaoning University of Traditional Chinese Medicine (2017). doi: 10.3969/j.issn.0411-8421.2021.06.017
28	G.Caminiti,2011	Tai Chi enhances the effects of endurance training in the rehabilitation of elderly patients with chronic heart failure. Rehabil. Res. Pract. 2011, 2011, 1–6
29	H.Zhou,2015	The effect of six-tips on cardiac function in patients with chronic heart failure. Yiayao Qianyan 2015, 5, 286–287.
30	G.Y.Yeh,2013	Tai Chi in patients with heart failure with preserved ejection fraction. Congest. Heart Fail. 2013, 19, 77–84
31	B.Zhou,2020	Effects of Taijiquan and cardiac rehabilitation exercise training on heart function, depression and sleep quality in patients with heart failure. J. Int. Psychiatry 2020, 47, 1016–1018, 1031.
32	X.J.Yang,2015	The effect of traditional Chinese exercises on exercise tolerance in patients with chronic heart failure. Chin. J. Nurs. 2015, 50, 193–197.
33	X.Yan,2016	Effect of Six tips and Baduanjin training exercise on exercise tolerance in patients with chronic heart failure. Chin. Med. Mod. Distance Educ. China 2016, 14, 126–128
34	J.H.Ke,2020	Effects of Baduanjin exercise on ventricular remodeling and cardiac function in patients with chronic heart failure. Chin. Med. Mod. Distance Educ. China 2020, 18, 74–76.
35	D.Wei,2003	Effect of simplified Taijiquan exercise on improving cardiac function in patients with heart failure
36	D.Yu,2019	"Effects of Taijiquan and Baduanjin on exercise tolerance and quality of life in patients with chronic heart failure," Journal of New Chinese Medicine, vol. 51, no. 3, pp. 274–277, 2019, in Chinese.
37	J.Yu,2015	Research on the effect of TCM calisthenics exercise on endurance in patients with chronic heart failure. Chin. Med. Mod. Distance Educ. China 2015, 13, 12–14.
38	M.L.Yu,2020	Application of Tai Ji Quan exercise in heart rehabilitation for elderly patients with heart failure after myocardial infarction. Chin. J. Rehabil. Theory Pract.
39	Z.H.Zhang,2021	Effect of Taijiquan on cardiopulmonary function and aldosterone level in elderly patients with chronic heart failure
40	G.Y.Yeh,2004	Effects of tai chi mind-body movement therapy on functional status and exercise capacity in patients with chronic heart failure: A

		randomized controlled trial. Am. J. Med. 2004, 117, 545–548.
41	G.Y.Yeh,2008	Enhancement of sleep stability with Tai Chi exercise in chronic heart failure: Preliminary findings using an ECG-based spectrogram method. Sleep Med. 2008, 9, 527–536.
42	G.Y.Yeh,2011	Tai Chi exercise in patients with chronic heart failure: A randomized clinical trial. Arch. Intern. Med. 2011, 171, 750–757.
43	L.S.Redwine,2019	A randomized study examining the effects of mild-to-moderate group exercises on cardiovascular, physical, and psychological well-being in patients with heart failure. J. Cardiopulm. Rehabil. Prev. 2019, 39, 403–408.
44	L.Hägglund,2018	A mixed methods study of Tai Chi exercise for patients with chronic heart failure aged 70 years and older. Nurs. Open 2018, 5, 176–185.
45	T.Yu,2019	The influence of Tai Chi rehabilitation program with different training time on cardiac function of patients with chronic heart failure. Chin. J. Integr. Med. Cardio/Cerebrovasc. Dis. 2019, 17, 1772–1775.
46	L.M.Deng,2021	Analysis of intervention effect of Baduanjin on patients with chronic heart failure. Shenzhen J. Integr. Tradit. Chin. West. Med. 2021, 31, 59–61.
47	Z.L.Kang,2021	Analysis of the effect of Baduanjin combined with aerobic endurance training on elderly patients with stable coronary heart disease complicated with chronic heart failure. Chin. J. Front. Med. Sci. (Electron. Version) 2021, 13, 39–42.
48	X.D.Deng,2018	Effect of Taichi on heart function and psychological state in MI patients with heart insufficiency. J. North Sichuan Med. Coll. 2018, 33, 545–547.
49	X.F.Pan,2016	Influence of Taijiquan exercise on cardiac function and quality of life in patients with chronic heart failure. Chin J Phys Med Rehabil. (2016) 38:51–3. doi: 10.1177/1043659610395770
50	L.Sang,2015(a)	Effect of Tai Chi rehabilitation exercises on heart function and quality of life in elderly patients with chronic heart failure of coronary heart disease. Chin. J. Gerontol. 2015, 35, 3957–3958.
51	Y.X.Zhou,2021	Effect of Baduanjin exercise on cardiac function and quality of life in patients with heart failure after myocardial infarction. China Morden Med. 2021, 28, 73–75, 79.
52	L.Sang,2015(b)	Effect of Tai Chi rehabilitation exercise on plasma angiotensin II and brain natriuretic peptide levels in elderly patients with chronic heart failure of coronary heart disease. Chin. J. Gerontol. 2015, 35, 4599–4600.
53	C.Li,2015	Effect of exercise tolerance in patients with chronic heart failure exercise training. Nurs. Pract. Res. 2015, 12, 36–37
54	L.H.Yuan,2016	Effect of Tai Chi on improvement of depression, sleeping quality and quality of life in elderly patients with chronic congestive heart

		failure complicated with depression. Guangxi Med. J. 2016, 38, 1547–1550.
55	N.Wang,2011	The Research on Chronic Heart Failure by Taijiquan With Drugs. Nanjing: Nanjing University of Chinese Medicine (2011).
56	L.Yuan,2017	The Influence of Taijiquan on Elderly Stability Patients with Chronic Congestive Heart Failure. Nanjing: Nanjing University of Chinese Medicine (2017).
57	F.Ding,2018	Observation on the curative effect of taiji yangshenggong combined with acupoint massage in treating patients with mild and moderate chronic heart failure. Chin J Phys Med Rehabil. (2018) 40:789–90.
58	D.E.Borrow,2007	An evaluation of the effects of tai chi chuan and chi kung training in patients with symptomatic heart failure: a randomised controlled pilot study. Postgrad Med J. 2007;83(985):717–721.
59	C.Huang,2014	"Study on the rehabilitation of cardiac function in patients with chronic heart failure (NYHA class III) with coronary heart disease," Master thesis in Chinese, Fujian University of Traditional Chinese Medicine, Fuzhou, China, 2014.
60	L.S.Redwine,2012	"A pilot study exploring the effects of a 12-week T'ai Chi intervention on somatic symptoms of depression in patients with heart failure," The Journal of Alternative and Complementary Medicine, vol. 18, no. 8, pp. 744–748, 2012.
61	Xiong XH,2017	Effect of BaDuanJin on cognitive function of patiens with coronary heart disease complicated with chronic heart failure," Clinical Medical & Engineering, vol. 24, no. 12, pp. 1723-1724, 2017.
62	Z.Shi,2018	"Clinical study of Baduanjin on rehabilitation treatment of patients with chronic heart failure," Master thesis in Chinese, Liaoning University of Traditional Chinese Medicine, Shenyang, China, 2018.
63	R.Zheng,2018	Chan-Chuang Qigong improves exercise capacity, depression, and quality of life in patients with heart failure," Hu Li Za Zhi, vol. 65, no. 5, pp. 34–44, 2018.
64	X.X.Li,2017	Analysis of the Therapeutic Effect of Hourly Movement Therapy in the Treatment of Chronic Heart Failure
65	S.Sato,2010	.Effect of Tai Chi training on baroreflex sensitivity and heart rate variability in patients with coronary heart disease. Int Heart J, 2010, 51(4): 238-241.
