# Supplementary material for: The Efficacy of Traditional Chinese Exercises in Patients With Chronic Heart Failure: An Umbrella Review and Meta-Analysis
Source: Rev Cardiovasc Med. 2026 Mar 20;27(3):46055. doi: 10.31083/RCM46055 (PMC13036533; doi:10.31083/RCM46055)
Supplement: Supplementary file 1 [file 2153-8174-27-3-46055-s1.zip › Supplementary Table 6 - ROBIS for risk of bias assessment.pdf]

Supplementary Table 4: Results of the ROBIS assessments.

Author, year (Country)	Phase 1	Phase 2				Phase 3
	Assessing relevance	Domain 1: study eligibility criteria	Domain 2: identification and selection of studies	Domain 3: collection and study appraisal	Domain 4: synthesis and findings	Risk of bias in the review
W.Y.Yang, 2023 (CHN)	√	√	×	√	√	√
B.W.Mei, 2023 (CHN)	√	√	×	√	√	√
M.Q.Dai, 2023 (CHN)	√	√	×	√	√	√
Q.Y.Bao, 2023 (CHN)	√	√	×	√	√	√
J.Q.Hui, 2022 (CHN)	√	√	×	√	√	√
F.Yao, 2021(CHN)	√	√	×	√	√	√
T.P.Ruth, 2020 (USA)	√	√	×	×	×	×
Y.Liao, 2020(CHN)	√	√	×	√	√	√
X.K.Chen, 2020(CHN)	√	√	×	√	×	×
A.Z.Wang, 2020 (CHN)	√	√	×	√	√	√
J.C.Li, 2018 (CHN)	√	√	×	√	×	×
H.Y.Wei, 2017 (CHN)	√	√	×	√	×	×
X.M.Ren ,2017 (CHN)	√	√	×	√	√	√
Q.Gu, 2017 (CHN)	√	√	×	√	√	√
L.Pan, 2013 (CHN)	√	√	×	√	√	√

Notes: √: low risk; ×: high risk.
