# Supplementary material for: The Efficacy of Traditional Chinese Exercises in Patients With Chronic Heart Failure: An Umbrella Review and Meta-Analysis
Source: Rev Cardiovasc Med. 2026 Mar 20;27(3):46055. doi: 10.31083/RCM46055 (PMC13036533; doi:10.31083/RCM46055)
Supplement: Supplementary file 1 [file 2153-8174-27-3-46055-s1.zip › Supplementary Table 7 - PRISMA checklist.pdf]

Supplementary Table 5: Results of the PRISMA checklist.

Section and topic		Items	W.Y.Yang, 2023 (CHN)	B.W.Mei, 2023 (CHN)	M.Q.Dai, 2023 (CHN)	Q.Y.Bao, 2023 (CHN)	J.Q.Hui, 2022(CHN)	F.Yao, 2021(CHN)	T.P.Ruth, 2020 (USA)	Y.Liao, 2020(CHN)	X.K.Chen, 2020(CHN)	A.Z.Wang, 2020 (CHN)	J.C.Li, 2018 (CHN)	H.Y.Wei, 2017 (CHN)	X.M.Ren, 2017 (CHN)	Q.Gu, 2017 (CHN)	L.Pan, 2013 (CHN)	**The percentage of "Y" (%)**
Title	Title	1	Y	Y	Y	Y	Y	Y	Y	Y	Y	Y	Y	Y	Y	Y	Y	100.00%
Abstract	Abstracts checklist	2	Y	Y	Y	Y	Y	Y	Y	Y	N	Y	Y	Y	Y	Y	Y	93.33%
Introduction	Rationale	3	Y	Y	Y	Y	Y	Y	Y	Y	Y	Y	Y	Y	Y	Y	Y	100.00%
	Objectives	4	Y	Y	Y	Y	Y	Y	Y	Y	Y	Y	Y	Y	Y	Y	Y	100.00%
Methods	Eligibility criteria	5	Y	Y	Y	Y	Y	Y	Y	Y	Y	Y	Y	Y	Y	Y	Y	100.00%
	Information sources	6	Y	Y	Y	Y	Y	Y	Y	Y	Y	Y	Y	Y	Y	Y	Y	93.33%
	Search strategy	7	Y	Y	Y	Y	Y	N	Y	Y	Y	Y	Y	Y	N	N	N	73.33%
	Selection process	8	Y	Y	Y	Y	Y	Y	Y	Y	Y	Y	Y	Y	Y	Y	Y	100.00%
	Data collection process	9	Y	Y	Y	Y	Y	Y	Y	Y	Y	Y	Y	Y	Y	Y	Y	100.00%
	Data items	10 (a)	Y	Y	Y	Y	Y	Y	Y	Y	Y	Y	Y	Y	Y	Y	Y	100.00%
		10 (b)	Y	Y	Y	Y	Y	Y	Y	Y	Y	Y	Y	Y	Y	Y	Y	100.00%
	Study risk of bias assessment	11	Y	Y	Y	Y	Y	Y	N	Y	Y	N	Y	Y	Y	Y	Y	86.67%
	Effect measures	12	Y	Y	Y	Y	Y	Y	N	Y	Y	Y	Y	Y	Y	Y	Y	93.33%
	Synthesis methods	13 (a)	Y	Y	Y	Y	Y	Y	Y	Y	Y	Y	Y	Y	Y	Y	Y	100.00%
		13 (b)	Y	Y	Y	Y	Y	Y	Y	Y	Y	Y	Y	N	Y	Y	Y	93.33%
		13 (c)	Y	Y	Y	Y	Y	Y	Y	Y	Y	Y	Y	Y	Y	Y	Y	100.00%
		13 (d)	Y	Y	Y	Y	Y	Y	Y	Y	Y	Y	Y	Y	Y	Y	Y	100.00%
		13 (e)	Y	Y	Y	Y	Y	Y	N	Y	Y	Y	Y	Y	Y	Y	Y	93.33%
		13 (f)	Y	Y	N	Y	Y	N	N	N	Y	N	Y	Y	Y	Y	Y	66.67%
	Reporting bias assessment	14	Y	Y	Y	N	Y	Y	Y	Y	Y	N	Y	Y	Y	Y	Y	80.00%
	Certainty assessment	15	Y	Y	N	Y	Y	N	N	N	Y	N	N	N	N	Y	Y	46.67%
Result	Study selection	16 (a)	Y	Y	Y	Y	Y	Y	Y	Y	Y	Y	Y	Y	Y	Y	Y	100.00%
		16 (b)	N	N	N	N	N	N	N	N	N	N	N	N	N	N	N	0%
	Study characteristics	17	Y	Y	Y	Y	Y	Y	Y	Y	Y	Y	Y	Y	Y	Y	Y	100.00%
	Risk of bias in studies	18	Y	Y	Y	Y	Y	Y	Y	Y	Y	Y	Y	Y	Y	Y	Y	100.00%
	Results of individual studies	19	Y	Y	Y	Y	Y	Y	Y	Y	Y	Y	Y	Y	Y	Y	Y	100.00%
	Results of syntheses	20 (a)	Y	Y	Y	Y	Y	Y	Y	Y	Y	Y	Y	Y	Y	Y	Y	100.00%
		20 (b)	Y	Y	Y	Y	Y	Y	Y	Y	Y	Y	Y	Y	Y	Y	Y	100.00%
		20 (c)	Y	Y	Y	Y	Y	Y	N	N	Y	Y	N	Y	Y	Y	Y	80.00%
		20 (d)	Y	Y	N	Y	Y	N	N	N	Y	N	N	Y	Y	Y	Y	60.00%
	Reporting biases	21	Y	Y	N	Y	Y	Y	N	Y	Y	Y	Y	N	Y	Y	Y	80.00%
	Certainty of evidence	22	Y	N	N	Y	Y	N	N	N	Y	N	N	N	N	N	N	26.67%
Discussion	Discussion	23 (a)	Y	Y	Y	Y	Y	Y	Y	Y	Y	Y	Y	Y	Y	Y	Y	100.00%
		23 (b)	Y	Y	Y	Y	Y	Y	Y	Y	Y	Y	Y	Y	Y	Y	Y	100.00%
		23 (c)	Y	Y	Y	Y	Y	Y	Y	Y	Y	N	Y	Y	Y	Y	Y	93.33%
		23 (d)	Y	Y	Y	Y	Y	Y	Y	Y	Y	Y	Y	Y	Y	Y	Y	100.00%
Other information	Registration and protocol	24 (a)	Y	N	Y	Y	Y	N	N	N	Y	N	N	N	N	N	N	33.33%
		24(b)	Y	N	Y	Y	Y	N	N	N	N	N	N	N	N	N	N	26.67%
		24 (c)	N	N	N	N	N	N	N	N	N	N	N	N	N	N	N	0%
	Support	25	Y	Y	N	Y	Y	Y	N	Y	Y	N	N	N	Y	Y	N	60.00%
	Competing interests	26	Y	N	Y	Y	Y	Y	N	Y	Y	N	N	N	N	Y	N	53.33%
	Availability of data, code and other materials	27	N	N	Y	Y	Y	N	N	Y	Y	N	N	N	N	Y	N	40.00%

Notes: Y: yes; N: no.
