# Supplementary material for: The Efficacy of Traditional Chinese Exercises in Patients With Chronic Heart Failure: An Umbrella Review and Meta-Analysis
Source: Rev Cardiovasc Med. 2026 Mar 20;27(3):46055. doi: 10.31083/RCM46055 (PMC13036533; doi:10.31083/RCM46055)
Supplement: Supplementary file 1 [file 2153-8174-27-3-46055-s1.zip › Supplementary Table 8 - Egger' test and funnel plot.pdf]

Supplementary Table 6: Results of Egger's test and Funnel plots.

Egger's test

	Number of studies	t-statistic	Degrees of freedom	*p*
**6-MWT**	**36**	-0.37	34	0.72
**MLHFQ**	**32**	-0.90	30	0.37
**LVEF**	**27**	1.14	25	0.26
**NT-proBNP**	**9**	-	-	-
**BNP**	**10**	0.13	8	0.90
**VO_2 max**	**12**	0.71	10	0.49
**AT**	**7**	-	-	-
**METs**	**9**	-	-	-

Funnel plots

(1) 6-MWT

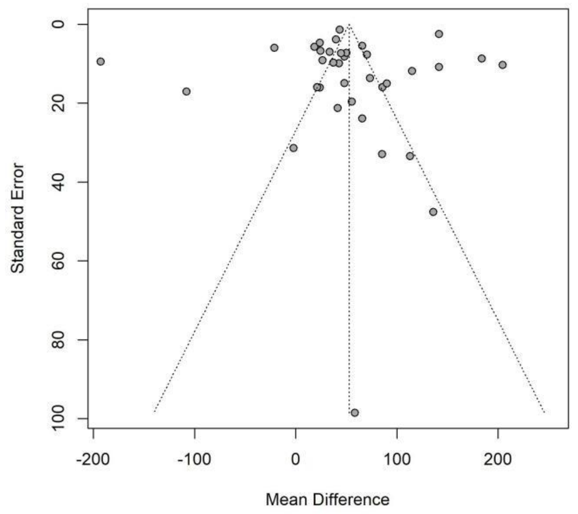

(2) MLHQF

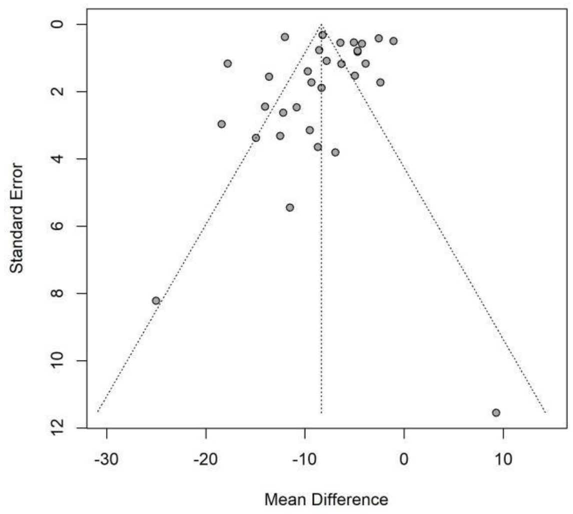

(3) LVEF

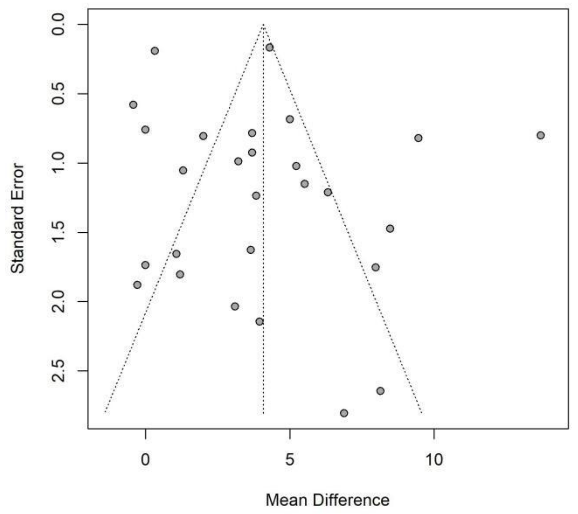

(4) NT-proBNP

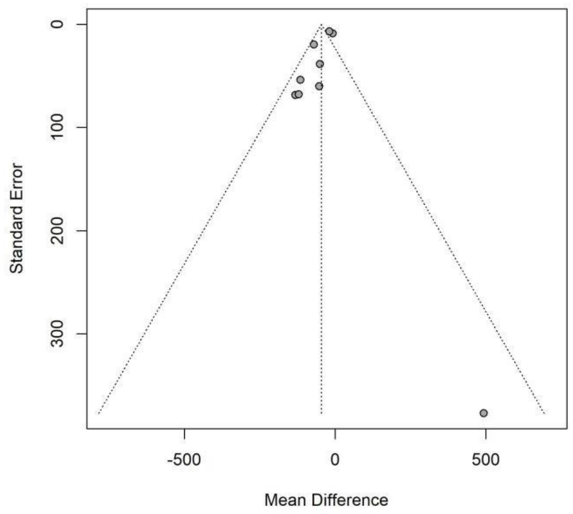

(5) BNP

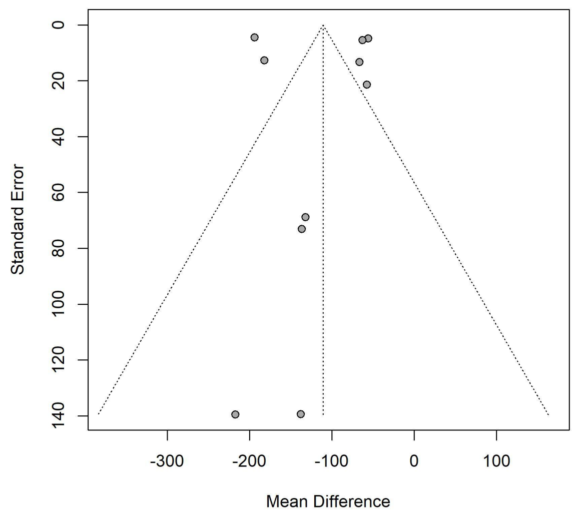

(6) VO_2 max

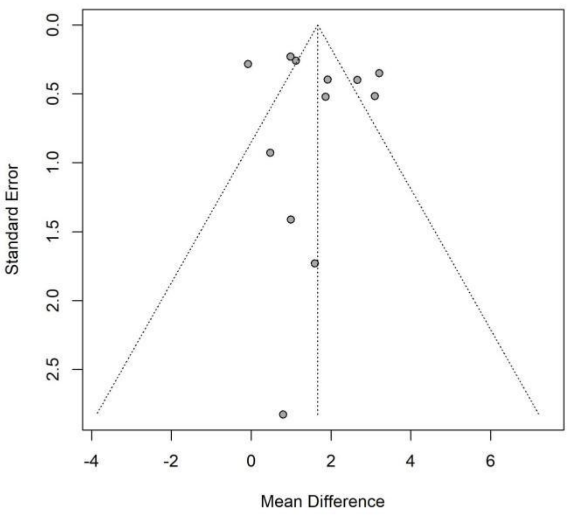

Notes:

6-MWT, 6 min-walk distance; MLHFQ, Minnesota Living with Heart Failure Questionnaire; LVEF, Left Ventricular Ejection Fraction; BNP, Nt-proBNP, N-terminal pro-B-type natriuretic peptide; B-type natriuretic peptide; VO2 max, Maximal Oxygen Consumption; AT, Anaerobic Threshold; METs, Metabolic Equivalents

It is typically recommended to have at least 10 or more independent studies to conduct the Egger's test.
