# Supplementary figures and images for: The Efficacy of Traditional Chinese Exercises in Patients With Chronic Heart Failure: An Umbrella Review and Meta-Analysis
Source: Rev Cardiovasc Med. 2026 Mar 20;27(3):46055. doi: 10.31083/RCM46055 (PMC13036533; doi:10.31083/RCM46055)

## Supplementary Table 7      Sensitivity Analyses

### (1) 6-MWT

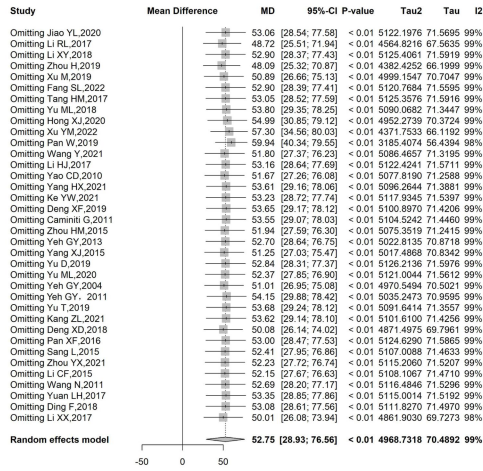

### (3) LVEF

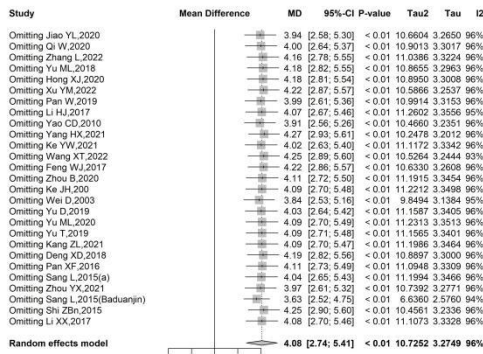

### (5) BNP

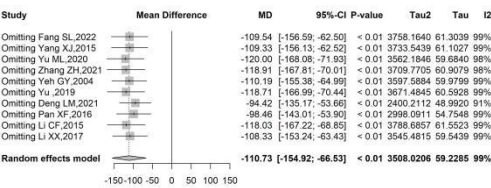

### (7) AT

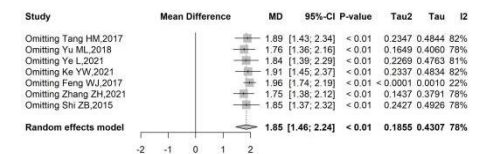

### (2) MLHQF

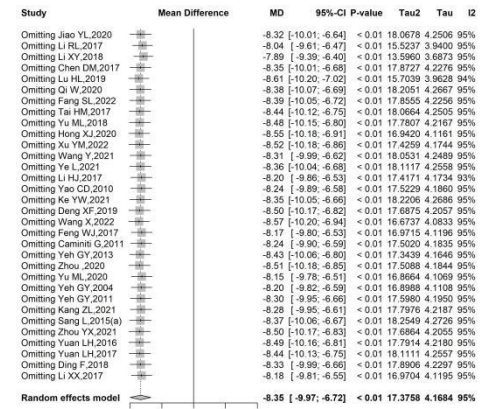

Supplement: Supplementary file 1 [file 2153-8174-27-3-46055-s1.zip › Supplementary Table 9 - sensitivity analyses.pdf]
